# Resolving Complex K–Pt–Sn Interactions in PtSn@K-MFI Catalysts for Alkane Dehydrogenation

**DOI:** 10.1021/jacs.5c01536

**Published:** 2025-04-03

**Authors:** Adrián Martínez Gómez-Aldaraví, Reisel Millán, Isabel Millet, Aroa Alós, Alejandro Vidal-Moya, Randall J. Meyer, Cristina Martínez, Avelino Corma, Mercedes Boronat, Pedro Serna, Manuel Moliner

**Affiliations:** † Instituto de Tecnología Química, Universitat Politècnica de València-Consejo Superior de Investigaciones Científicas, Avenida de los Naranjos s/n, València 46022, Spain; ‡ ExxonMobil Technology and Engineering Co., Annandale, New Jersey 08801, United States

## Abstract

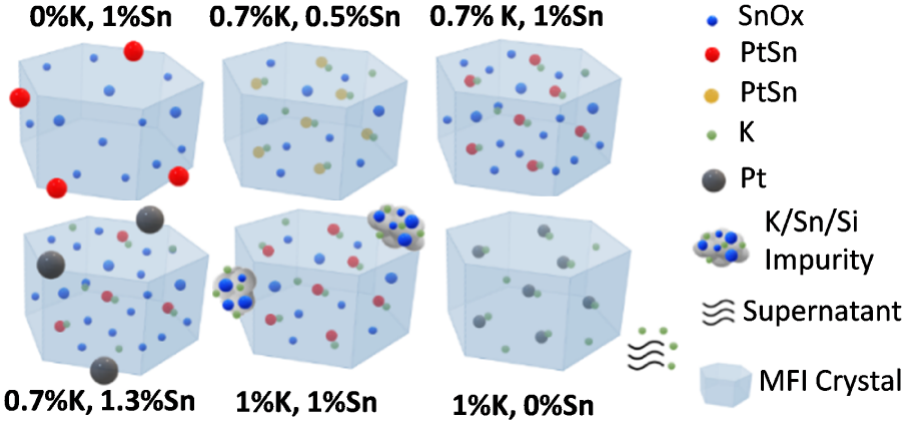

K and Sn contents were rationalized during the synthesis of PtSn@K-MFI to maximize metal dispersion and stability along the MFI crystallites. Experimental results and theoretical calculations reveal a stoichiometry of ∼1 K per unit cell of MFI, limiting then the final K incorporation within siliceous MFI crystals at ∼0.7 wt %. Above this stoichiometry, K is not incorporated into the final solids unless significant amounts of Sn are simultaneously present, leading to the formation of tin-silicate precipitates. The optimized PtSn@K-MFI catalysts improve the catalytic performance of well-established references, as PtSn/SiO_2_, for the propane dehydration (PDH) reaction. In particular, low Sn loadings (below 0.5 wt %) result in higher time-on-stream (TOS) deactivation catalytic profiles but excellent regenarability after consecutive PDH reaction, while higher Sn content (close to 1 wt %) minimizes TOS deactivation due to the maximization of Pt–Sn bonds but consecutive regenerations result in significant metal sintering. Increasing Sn contents within MFI crystallites facilitates Pt sintering and, thus, occurring catalyst deactivation upon regeneration cycles. As a result of complex interconnected nucleation/crystallization processes, fine-tuning rationalizations of one-pot synthesis approaches can substantially influence the final atomic and subnanometric metal interactions and, consequently, the catalytic and sintering-resistance properties when exposed to highly demanding industrial conditions.

## 1 Introduction

In the past decade, the availability of low-cost C_2_–C_4_ light alkanes has abruptly raised with the increase in shale gas production,^1^ providing new opportunities to commodity chemicals outside conventional syngas and steam cracking processes.^2,3^ Among the numerous routes to selectively turn light alkanes into useful building blocks, their direct dehydrogenation to olefins is an industrial reality, thanks to highly active and selective commercial catalysts that make the overall operation profitable.^4−6^ Nevertheless, thermodynamic limitations of this transformation impose high reaction temperatures to reach the conversion target, making the process energy intensive (i.e., burning of natural gas in external heaters), while triggering nontrivial catalyst deactivation challenges that negatively impact both economic and environmental metrics.^3,5^

Supported-Pt catalysts have been broadly investigated to promote the direct dehydrogenation of light alkanes to olefins, mainly propane to propylene, due to their remarkable ability to activate C–H bonds, while largely avoiding C–C bond cleavage.^7,8^ Sn is a typical, necessary, Pt promoter that further reduces undesired side transformations, such as hydrogenolysis and coke formation reactions.^7−9^ The main disadvantage of these common supported-metal catalysts is that they suffer permanent deactivation when subjected to severe redox stress, for example, during reaction and coke burn regeneration cycles in typical PDH processes, which causes extensive sintering of the metal nanoparticles.^10,11^ Because of this deactivation problem, commercial PtSn catalysts must undergo complex continuous catalyst rejuvenation protocols (e.g., oxychlorination), which operate under increased cost, safety controls, and waste management regulations, to redisperse the metal species and preserve proper levels of catalytic activity.^12^

A recent strategy to boost the stability of metal nanoparticles has been the encapsulation of subnanometric PtSn clusters within the pores of pure silica MFI zeolite, following a one-pot synthesis approach that relies on the use of potassium cations to introduce negative charge into the zeolite framework, in the absence of tetravalent elements (e.g., Al).^13,14^ HAADF-STEM and i-DCP imaging of the resultant PtSn@K-MFI catalyst demonstrated the regioselective stabilization of subnanometric PtSn clusters (∼0.6–0.8 nm) in the sinusoidal 10-ring channels of MFI, associated with the formation of silanol/siloxy nests with potassium cations located at sinusoidal channels that connect with straight 10-ring pores.^15^ As a result of these zeolite binding sites, outstanding resistance against metal sintering was observed compared to other zeolite-based catalysts prepared by conventional impregnation methods.^13,14^ The resultant catalyst was not only sintering-resistant but could also catalyze the dehydrogenation of light alkanes to olefins with minimal cracking and coke formation (i.e., minimal time-on-stream deactivation). Other bimetallic clusters, such as PtZn, PtGe, or PtIn, have been encapsulated within pure silica zeolite structures, as active and stable catalysts for dehydrogenation of light alkanes.^16−18^

Preliminary inspection of the influence of the Sn content in the original PtSn@K-MFI formulations revealed an optimal Sn content of ∼0.9 wt %, and the requirement of long reduction treatments to properly activate the catalyst in H_2_ prior to the PDH reaction (i.e., reduction times longer than 12 h).^14^ In this former study, lowering the Sn content to 0.4 wt %, or lowering the activation times to <12 h, led to a significant increase in the time-on-stream (TOS) deactivation rates, presumably as a result of decreased Pt–Sn interactions in the final material.^14^

The present work was motivated by these results and previous observations that one-pot synthesis of Sn- and Ti-containing zeolites can be problematic when alkali cations are simultaneously present, because the latter tend to induce the formation of insoluble tin- or titano-silicates,^19,20^ thus altering the amount of metal available, in practice, inside the zeolite.

Keeping this in mind, we decided to systematically investigate how the nominal composition of PtSn@K-MFI catalysts affects the composition, structure, and performance of the final materials. More specifically, we independently varied the K and Sn contents and characterized the resultant solids by powder X-ray diffraction (PXRD) and scanning electron microscopy (SEM), to evaluate the crystallinity and homogeneity of the zeolite particles, followed by careful assessment of the metal dispersion along the MFI crystallites by scanning transmission electron microscopy (STEM). Information obtained from fresh (unreacted) samples, blank experiments, and catalysts after the elimination of spectators (through carefully designed postsynthetic treatments), combined with theoretical calculations and ^1^H MAS NMR spectroscopy, are used to rationalize the maximum amount of K that can be inserted at framework defects of the siliceous MFI crystals (∼ 0.7 wt %). Above this stoichiometry, K is not incorporated into the final solids unless significant amounts of Sn are simultaneously present, which leads to the formation of tin-silicate precipitates. Below ∼0.7 wt % K in the final solid, insufficient silanol/siloxy density is achieved to properly stabilize subnanometric Pt species in either H_2_ or O_2_ at high temperatures, leading to the formation of large Pt aggregates.^21^

The performance of these catalysts was tested in the PDH reaction and compared with standard references. At low Sn loadings (approximately lower than 0.5 wt %), increased time-on-stream (TOS) deactivation is generally observed, but the excess of Sn is also detrimental, because it appears to displace part of the Pt outside the zeolite pores, where sintering and, thus, deactivation upon regeneration occurs. Problems caused by the excess of Sn inside the zeolite can be avoided by working above the maximum allowed K/zeolite stoichiometry (at ∼0.7 wt % K), which induces a fraction of the Sn to precipitate as a tin-silicate impurity. Catalysts made with different nominal gel compositions, but similar inside the MFI crystals, appear to behave similarly in the PDH reaction, in terms of activity, TOS deactivation, and regenerability, regardless of the presence or absence of surface tin-silicate impurities, which we infer act as mere spectators under the selected testing conditions.

X-ray absorption spectroscopy (XAS) characterization of optimized catalysts is used to confirm the correlation among the Sn present inside the zeolite, the number of Pt–Sn bonds after H_2_ treatments, and the TOS deactivation in the PDH reaction. Dropping the Sn content to 0.5 wt %, for example, results in decreased Pt–Sn bonds after 1 h in H_2_ at 600 °C compared to samples made at 1 wt % Sn, all other synthesis variables constant. Extending the reduction time of the low loading Sn sample to 12 h corrects this difference and leads to decreased TOS deactivation, consistent with previous results.^14^ However, the optimal PtSn@K-MFI formulation presented here achieves optimal selectivity and TOS deactivation after only 1 h reduction in H_2_, unlike previous samples, which requires more than 12 h to achieve analogous results.^14^ The performance of optimized catalysts in this work is also markedly superior relative to a PtSn/SiO_2_ reference.^22^

Our results highlight the complexity in the relationships between the synthesis conditions, the composition and structure of the final materials, and their catalytic performance in one-pot synthesis of metal-containing zeolites, particularly when the active sites involve several elements (in our case, K, Sn, and Pt) that must be adjusted to optimize multiple performance criteria simultaneously, such as activity, selectivity, TOS deactivation (i.e., coke), and stability against sintering in high-temperature redox processes.

## 2 Results and Discussion

### 2.1 One-Pot Synthesis of PtSn@K-MFI Samples and Postsynthetic Treatments with NaOH: Influence of K and Sn Contents

The original recipe to synthesize PtSn@K-MFI used a commercially available aqueous solution of tetrapropylammonium hydroxide (TPAOH) containing trace amounts of K in the solution.^13,14^ This prior work showed that the presence of K cations within the final PtSn@K-MFI solids is critical to stabilize subnanometric PtSn clusters in the sinusoidal MFI channels, which do not sinter when subjected to cyclic high-temperature H_2_/O_2_ treatments, unlike most other catalysts reported in the literature.^13,14^

Surprisingly, detailed FESEM and STEM analysis of these samples indicates that small amounts of precipitates (i.e., impurities) are present in the as-prepared materials, mixed with the MFI crystals, the abundance of which varies depending on the specific batch of TPAOH used, all other synthesis variables maintained constant (see experimental details for MFI-PtSn1 samples, and FESEM and STEM images in Figures S1 and S2, respectively). Energy-dispersive X-ray spectroscopy (EDX) shows that these regions are enriched in Sn relative to the average Sn composition in the final solids (spectra 1 and 2 in Figure S2), thus confirming the precipitation of part of the Sn as a tin-silicate phase.

We postulated that the precipitation of tin-silicates is promoted by the presence of K, whose concentration may vary slightly from batch to batch in commercial TPAOH sources. This hypothesis finds roots in previous works on TS-1 materials, where it was observed that K in the synthesis gel induces the precipitation of titano-silicates.^19,20^ The variability in results using different TPAOH batches suggests that the K, Sn, and/or Pt speciation might be affected by subtle differences in the synthesis gels within a narrow compositional range, which triggered this new investigation.

To better understand and control the formation of active sites in one-pot synthesis of PtSn@K-MFI catalysts, we explored preparations that use a commercially available K-free TPA solution and KCl as a more reliable K source (see experimental section S.1 for details). For this initial study, the initial K content in the synthesis gel was fixed at 1.3 wt %, whereas the Sn content was varied from 1.4 to 0.5 wt % (note: all weight percentages are on a SiO_2_ basis, MFI-PtSn2-5 in Table 1).

**Table 1:** ICP Analyses of the Different PtSn@K-MFI Samples Prepared by One-Pot Synthesis Methods (Note: The Weight Percentages Both in the Gel and Solids Are Silica Basis)

	Composition gels			Solids		
Sample	Pt (wt %)	K (wt %)	Sn (wt %)	Pt (wt %)	K (wt %)	Sn (wt %)
MFI-PtSn1a	0.40	n.d.^a^	1.00	0.41	0.88	1.00
MFI-PtSn1b	0.40	n.d.^a^	1.00	0.40	1.15	1.05
MFI-PtSn2	0.40	1.30	1.40	0.41	1.05	1.38
MFI-PtSn2_NaOH^b^	---	---	---	0.43	0.73	0.77
MFI-PtSn3	0.40	1.30	1.00	0.45	1.03	0.98
MFI-PtSn4	0.40	1.30	0.70	0.42	0.87	0.72
MFI-PtSn5	0.40	1.30	0.50	0.43	0.81	0.49
MFI-Pt6	0.40	1.30	---	0.43	0.65	---
MFI-7a	---	1.30	---	---	0.70	---
MFI-7b	---	---	---	---	---	---
MFI-PtSn8	0.40	0.65	1.30	0.44	0.60	1.24
MFI-PtSn9	0.40	0.65	1.00	0.46	0.67	1.08
MFI-PtSn10	0.40	0.65	0.50	0.48	0.60	0.54
MFI-PtSn11	0.40	0.65	0.20	0.41	0.58	0.19

a Nondetermined.

b Treated with 20 wt % NaOH aqueous solution at 60 °C for 1 h (liquid/solid ratio = 10).

**Figure 1:**
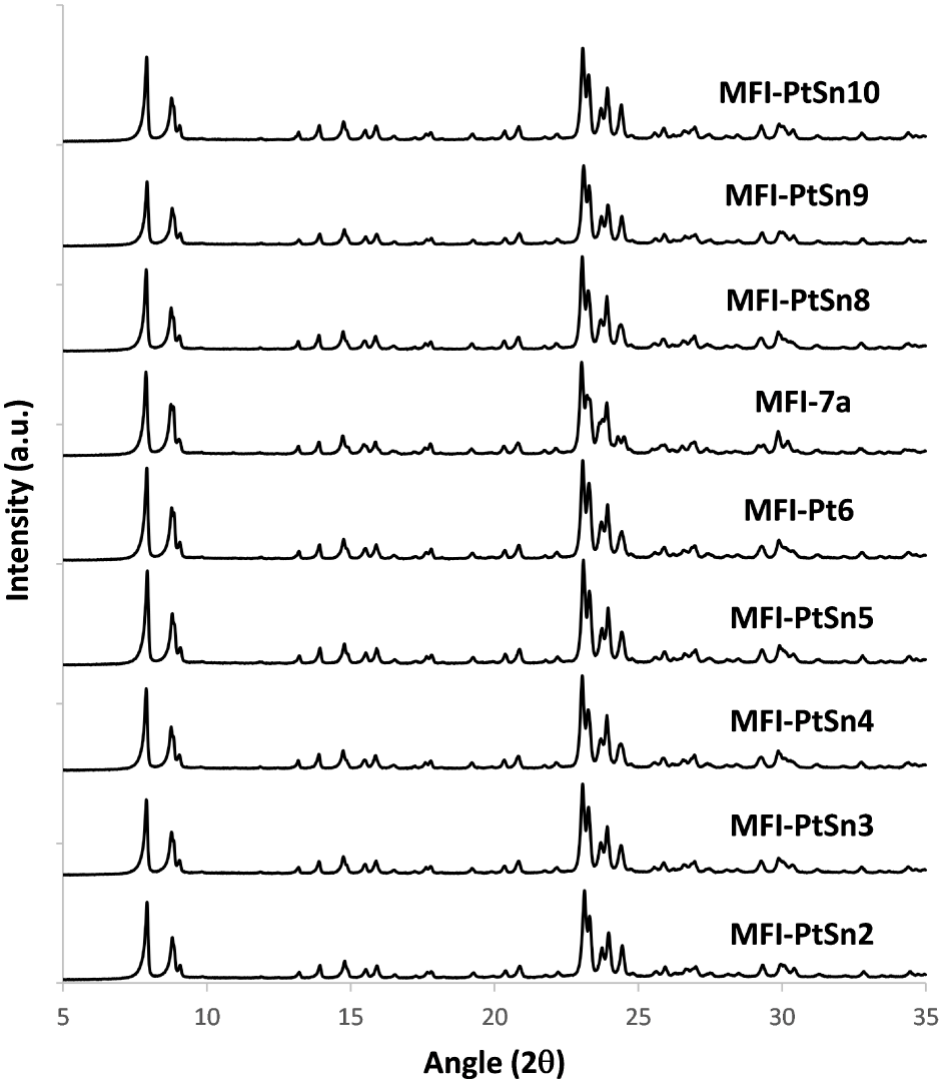
PXRD patterns of different PtSn@K-MFI samples in their calcined forms.

**Figure 2:**
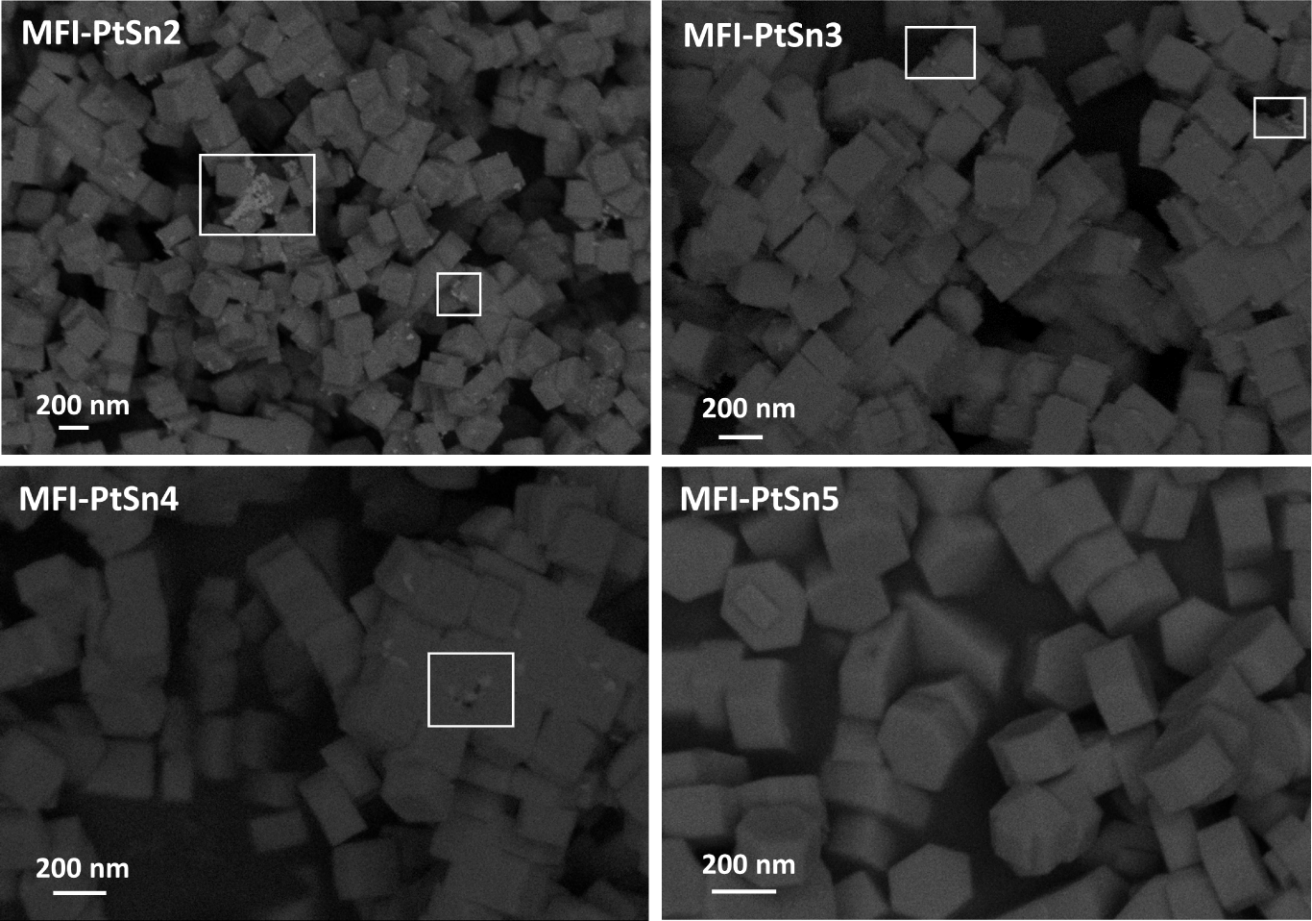
FESEM images of the as-prepared MFI-PtSn2-5 samples.

**Figure 3:**
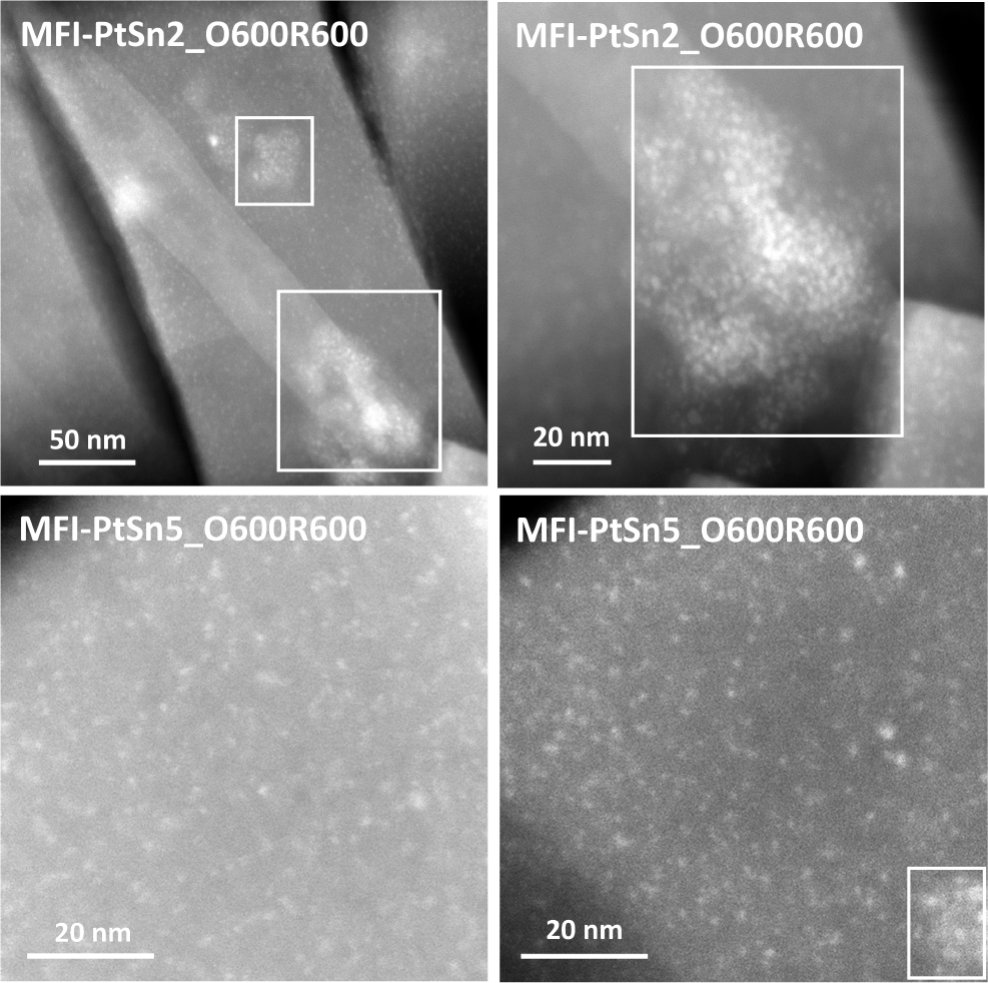
STEM images of the MFI-PtSn2 and MFI-PtSn5 samples after being subjected to calcination and reduction treatments at 600 °C in air and hydrogen, respectively (O600R600).

**Figure 4:**
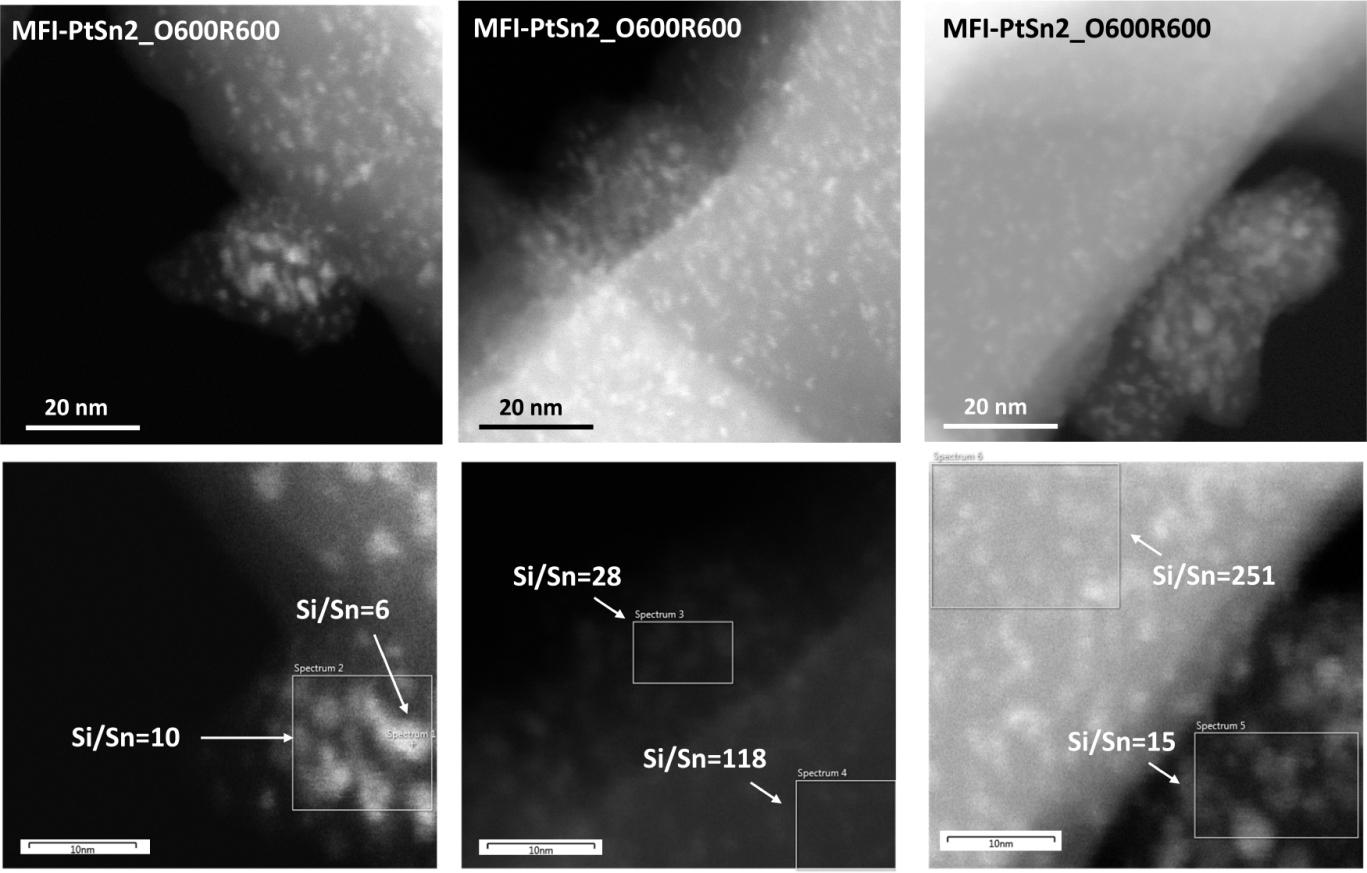
STEM images (top) and EDX/STEM analysis (bottom) of the MFI-PtSn2 sample after being subjected to calcination and reduction treatments at 600 °C in air and hydrogen, respectively (O600R600).

The PXRD patterns of these PtSn@K-MFI samples, after calcination, are consistent with the crystallization of the MFI structure (MFI-PtSn2-5 in Figure 1). The corresponding FESEM images confirm the formation of homogeneous zeolite crystallites with average sizes close to 200–300 nm (Figure 2). However, an amorphous contamination is also observed by FESEM in some cases (see squared areas in Figure 2), which becomes increasingly more prominent as the Sn content increases (thus, maximum at 1.4 wt % Sn in MFI-PtSn2 and virtually inappreciable at 0.5 wt % Sn in MFI-PtSn5). STEM-EDX analysis of samples calcined in air and reduced in hydrogen at 600 °C confirms the presence of these impurities in all samples, the size of which oscillates between ∼ 10 nm, thus too small to be observable by FESEM, when the amount of Sn is small (i.e., 0.5 wt % in MFI-PtSn5, Figure 3-bottom right), to >100 nm, when the amount of Sn is 1.4 wt % (MFI-PtSn2, Figure 3-top). Chemical analyses confirm, again, that these regions are Sn-enriched (Si/Sn < 20, compared to 110 in the zeolite crystals, Figure 4-bottom). The STEM images also show the formation of subnanometric metallic clusters in both materials,^23^ which appear preferentially located in areas of the MFI lacking other impurities (see Figure 3).

Interestingly, ICP analysis of samples MFI-PtSn2-5 shows that the Pt and Sn contents in the final solids are analogous to those introduced in the corresponding synthesis gels, but the K content was lower (0.8–1 wt % vs ∼1.3 wt % in the gels, MFI-PtSn2-5 in Table 1). Moreover, the amount of K incorporated decreased as the Sn content was reduced in the 1.4 to 0.5 wt % range. The relationship between these two variables is linear (Figure S3) and predicts that the maximum amount of K that incorporates into the final solid is 0.68 wt %, when Sn is not present (extrapolation of the trendline to Sn wt % = 0, i.e., *Y*-intercept at the origin in Figure S3). This observation, and the fact that the concentration of amorphous precipitates in the final solids becomes more significant as the amount of Sn increases, led us to infer that K cannot be incorporated into the solids above this threshold (∼0.68 wt %), unless it is sequestered by Sn into the tin-silicates phase (further evidence below).

We then designed an additional experiment to better understand the fate of K and Sn in these samples after developing a protocol that selectively removes inorganic impurities from the external surface of the zeolite, via controlled base-washing of uncalcined samples. For this experiment, as-prepared MFI-PtSn2 sample containing amorphous tin-silicate impurities was treated with 20 wt % NaOH aqueous solution at 60 °C for 1 h, at a fixed liquid/solid ratio of 10, leading to conditions alkaline enough to dissolve these nondesired impurities (see experimental section for further experimental details). The procedure does not remove the organic TPA inside the MFI pores, which we expected to protect the zeolite and any internal inorganic extra-framework cations from being reacted and extracted. FESEM images before and after these treatments demonstrate that the amorphous contamination on the surface of the MFI crystals is effectively removed through the process, without alteration of the zeolite particle morphology (see Figure S4-bottom). The disappearance of these amorphous impurities is, moreover, accompanied by a substantial decrease in the K and Sn contents (K content drops from 1.05 to 0.73 wt %, and Sn content drops from 1.38 to 0.77 wt %; see MFI-PtSn2 and MFI-PtSn2_NaOH in Table 1). The Pt content, in contrast, appears unaffected by the NaOH treatment (0.41 and 0.43 wt % for MFI-PtSn2 and MFI-PtSn2_NaOH, respectively, Table 1). These results confirm that the NaOH treatment removes the amorphous precipitates selectively, while maintaining metals inside the MFI crystals intact, thanks to the protecting role of TPA prior to calcination. The result also suggests that the vast majority of Pt is successfully encapsulated inside the zeolite pores during its crystallization.

Interestingly, the amount of K retained by the zeolite after the NaOH wash, once the vast majority of the tin-silicate has been removed, is very close to the maximum amount of K that is incorporated into the final solid, according to Figure S3, in experiments conducted at variable Sn concentrations in the synthesis gels. This observation is consistent with our hypothesis that samples that avoid tin-silicate impurities cannot incorporate K above a certain stoichiometric concentration of about ∼ 0.7 wt % in the final product.

Direct evidence for this postulation was provided through additional experiments run under Sn-free conditions using TGA, ^1^H 2D DQ/SQ NMR, and DFT calculations to rationalize the observed stoichiometry in a quantitative manner.

### 2.2 Sn-Free Pt@K-MFI Blank Experiments: Understanding the Required Stoichiometric K Content

In order to determine the maximum K content that can be incorporated through a one-pot method inside purely siliceous MFI crystals, we performed two more experiments that avoid Sn in the synthesis gels and, therefore, eliminate the possibility of forming a K-doped tin-silicate impurity. Both of these samples were synthesized at high nominal K contents (i.e., 1.3 wt %), but the first one targeted a nominal Pt content of 0.4 wt % (MFI-Pt6), whereas the second was a Pt-free sample (MFI-7a). Additionally, a K-free, Sn-free, Pt-free MFI sample, MFI-7b, was also prepared, to further evaluate if the K content has an influence on the particle size and/or morphology of the resultant zeolite crystals.^24^ As demonstrated by FESEM (see Figure S5), the MFI crystals show similar particle size and morphology regardless of the presence or absence of K. The loading of K in the final solids, measured by ICP, is similar in MFI-Pt6 and MFI-7a materials, ∼0.65–0.69 wt %, which is almost half the amount introduced in the synthesis gels, and is also significantly lower compared to syntheses run in the presence of Sn at equivalent nominal K values (Table 1, comparison between MFI-Pt6 and MFI-7a vs PtSn2-5). From these experiments, we estimate that the maximum amount of K that can be incorporated into the MFI crystals via a one-pot synthesis method is, again, ∼0.7 wt %, consistent with the prediction from Figure S3.

To understand the genesis of this limitation, we used elemental and thermogravimetric analyses and determined that the as-prepared K-MFI samples incorporate ∼4 molecules of TPA per unit cell of the zeolite, which contains ∼96 T atoms (Table S2). TPA, thus, is expected to occupy the four pore intersections per unit cell of the zeolite, in good agreement with conventional MFI syntheses. In the absence of tetravalent elements such as Al, having 4 TPAs per unit cell locates one framework defect in every intersection of the zeolite, just to counterbalance the positive charge of the extra-framework organic cation. At 0.7 wt % K, which is the maximum amount of K that we could experimentally introduce into the MFI crystals, materials are incorporating ∼1 K atom per unit cell, thus one extra framework defect every four intersections (Table S2).

**Figure 5:**
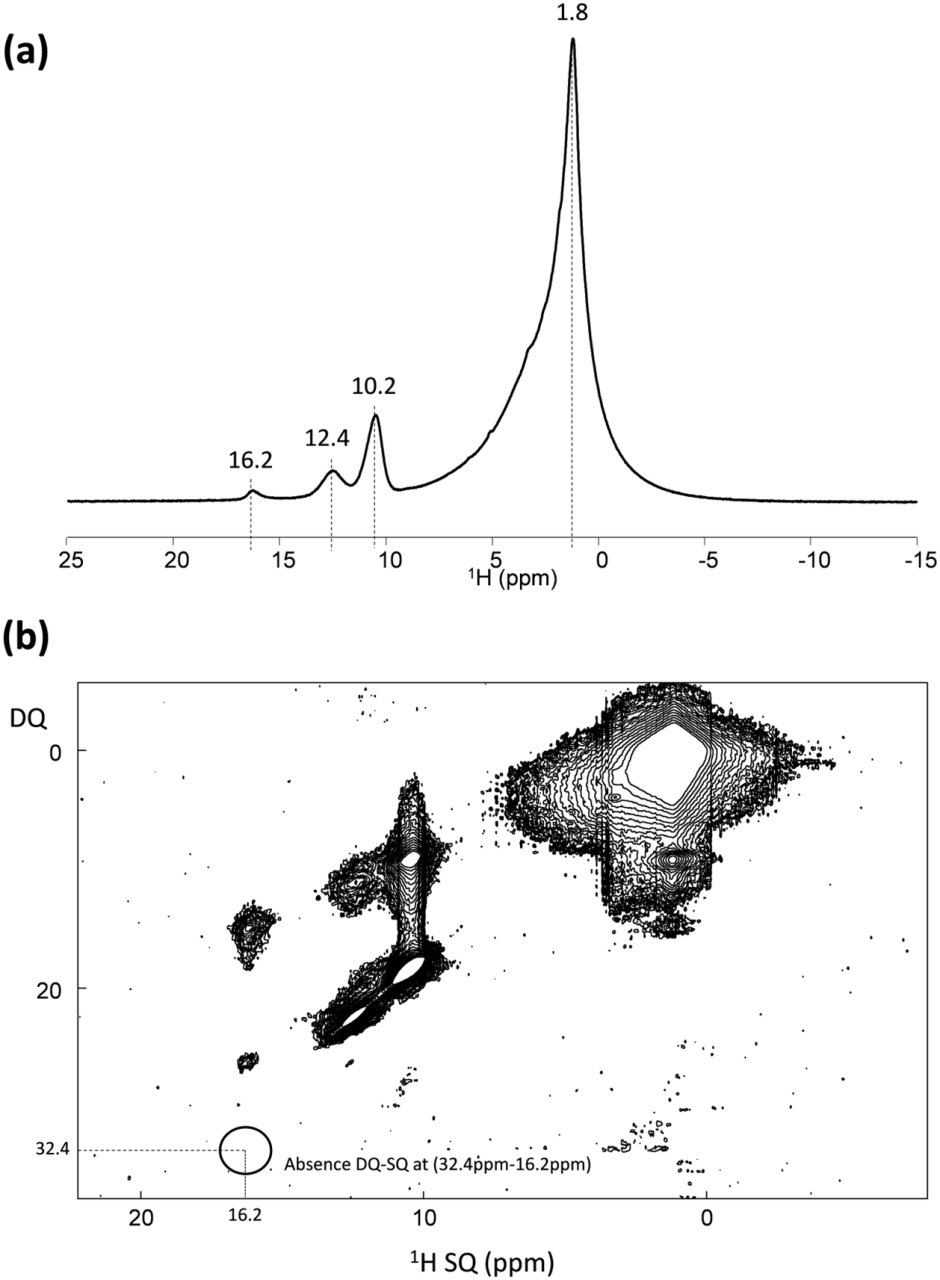
^1^H MAS NMR (a) and ^1^H DQ-SQ MAS NMR (b) spectra of the as-prepared MFI-7a sample.

The ^1^H MAS NMR spectrum of MFI-7a shows an asymmetric main signal centered at 2 ppm (see Figure 5a), whereas other smaller resonances appear at high field (10.2, 12.4, and 16.4 ppm, see Figure 5a). A detailed integration of the ^1^H MAS NMR spectrum reveals ∼112 protons for the signal centered at ∼2 ppm, ∼9 protons associated with the signal at 10.2 ppm, ∼3 protons for the signal at 12.4 ppm, and ∼1 proton for the signal at 16.4 ppm (see Figure 5a). The signal at 2 ppm has been assigned to protons of the methyl and methylene groups in TPA molecules (4 per MFI unit cell), whereas the signals at 10.2 and 12.4 ppm have been assigned to defective sites containing a Si-vacancy (see Figure S6a),^22^ compensating the positive charge of the organic TPA cation in the TPA-MFI zeolite framework. These signals account for 12 protons, corresponding to the presence of four (SiOH)_3_–SiO^–^ clusters per unit cell (see Figure S6a).^22^ Finally, to the best of our knowledge, the signal at 16.4 ppm, corresponding to ∼1 proton per unit cell, has not been described in the literature. It is worth noting that this signal does not appear if the MFI is prepared under K-free conditions (see Figure S7). To assign this signal, we performed ^1^H 2D DQ/SQ NMR experiments. Because no DQ correlation is observed at 32.8 ppm (2 × 16.4 ppm, see circle in Figure 5b), and because DQ signals require at least a pair of protons, we infer that the 16.4 ppm signal derives from a single proton. The low-field shift of this signal could suggest a strong hydrogen bond interaction, which is more pronounced than that observed in the (SiOH)_3_–Si-O^–^ cluster. To maintain the neutral charge of the TPA-MFI structure, we tentatively assign the signal at 16.4 ppm to a SiOH–SiO^–^ connectivity defect compensated by K^+^ (see Figure S6b), in good agreement with the presence of ∼1 K per MFI unit cell.

**Figure 6:**
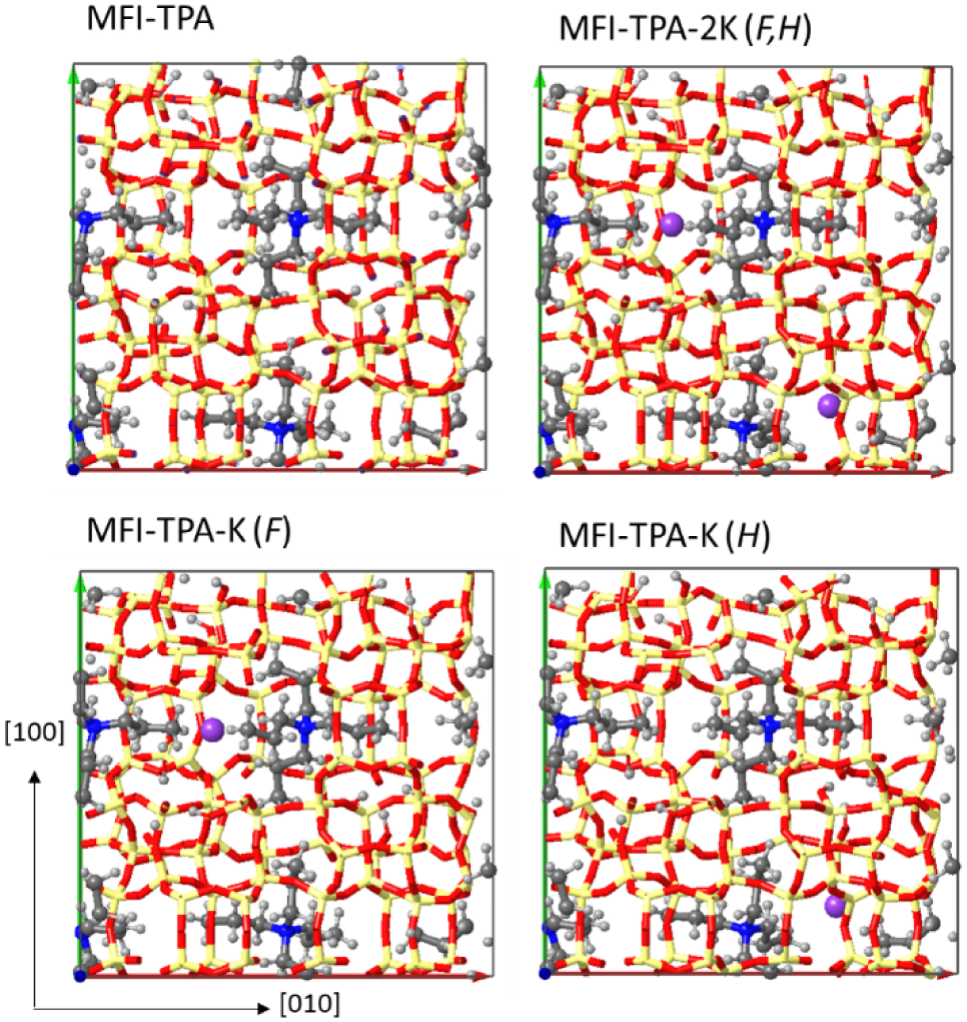
Optimized geometries of MFI-TPA, MFI-TPA-K, and MFI-TPA-2K models viewed from the [001] direction, showing the location of the TPA molecules at the intersections and the K cations in the sinusoidal channels. Framework Si and O atoms depicted as yellow and red sticks, N, C, H, and K atoms depicted as blue, gray, white, and pink balls.

Periodic DFT methods have been employed to calculate the energy necessary to add one K per unit cell to the MFI-TPA system, with four TPA molecules and four associated silanol/siloxy nests placed in the four intersecting channels of the MFI unit cell. The K^+^ cation and the associated OH^–^ counterion were placed in regions unoccupied by the TPA molecules, that is, around the pore intersections, in the sinusoidal channels, and in the lateral cages. K is too big to fit into the cages, and in the absence of TPA molecules, it is well stabilized in both types of channels (see relative energies for MFI-K models in Table 2). However, when the straight channels and the intersections are occupied by TPA molecules, K can be stabilized only in the sinusoidal channels (see relative energies for MFI-TPA-K models in Table 2). In the most stable systems, *F* and *H*, K is located in a sinusoidal channel and the associated OH is in a lateral cage forming an additional H bond (see Figures 6 and S8). In these two cases, the calculated energies to incorporate K are negative, indicating that the process is thermodynamically favorable.

**Table 2:** Relative Stability of MFI-K and MFI-TPA-K Models and Energy of Incorporation of K (*E*_inc_) in Different Locations within the MFI Unit Cell

	K location	OH location	E_rel_MFI-K (kcal/mol)	E_rel_MFI-TPA-K (kcal/mol)	*E*_inc_ (kcal/mol)
A	cage	sin	38.3	48.3	6.2
B	cage	str	34.8	50.7	12.1
C	str	sin	6.7	33.9	23.4
D	sin	str	8.1	22.6	10.7
E	str	cage	2.8	16.8	10.1
F	sin	cage	3.9	**2.0**	**–5.8**
G	cage	sin	34.5	41.4	3.1
H	sin	cage	1.9	**0.0**	**–5.7**
I	cage	str	39.3	47.9	4.8
J	str	sin	1.0	35.5	30.6
K	sin	sin	**0.0**	13.2	9.3
L	sin	sin	3.3	11.9	4.7

Interestingly, the simultaneous incorporation of two K cations, and the associated OH, is clearly endothermic in these two favorable positions, with a calculated *E*_inc_ value of 14.9 kcal/mol. Analysis of the optimized geometries of these systems (Figure 6) shows that the two K cations in the MFI-TPA-K2 model are separated by 11.974 Å and that neither the silicate framework nor the interaction of K with the surrounding O atoms is modified (see optimized Si–Si and K–O distances in Table S3). The energy penalty to add a second K in the same unit cell might therefore be attributed to unfavorable fitting of TPA molecules upon a small displacement within the pores, caused by steric and electrostatic TPA/K repulsions. This is consistent with the experimental observation that samples never incorporate K above a 1:1 unit cell stoichiometry, unless other forms of species such as tin-silicates can be formed, in synthesis made with large amounts of Sn present.

### 2.3 Synthesis and Characterization of PtSn@K-MFI Materials at Stoichiometric K Contents

Next, we decided to investigate the properties of new PtSn@K-MFI materials made with a stoichiometric content of K in the synthesis gels, corresponding to the maximum amount of K that the MFI structure can theoretically adsorb without the precipitation of tin-silicates (∼0.7 wt % K, *vide supra*). In other words, these syntheses aimed at locating all added K inside the MFI crystals, a result that, presumably, should happen even if Sn was present in a large excess (as per the base-washing experiments depicted in Section 2.1). For this study, 0.7 wt % K was fixed in the gels, and three Sn contents were evaluated, at 1.3, 1.0, and 0.5 wt % (MFI-PtSn8, MFI-PtSn 9, and MFI-PtSn10 in Table 1, respectively).

**Figure 7:**
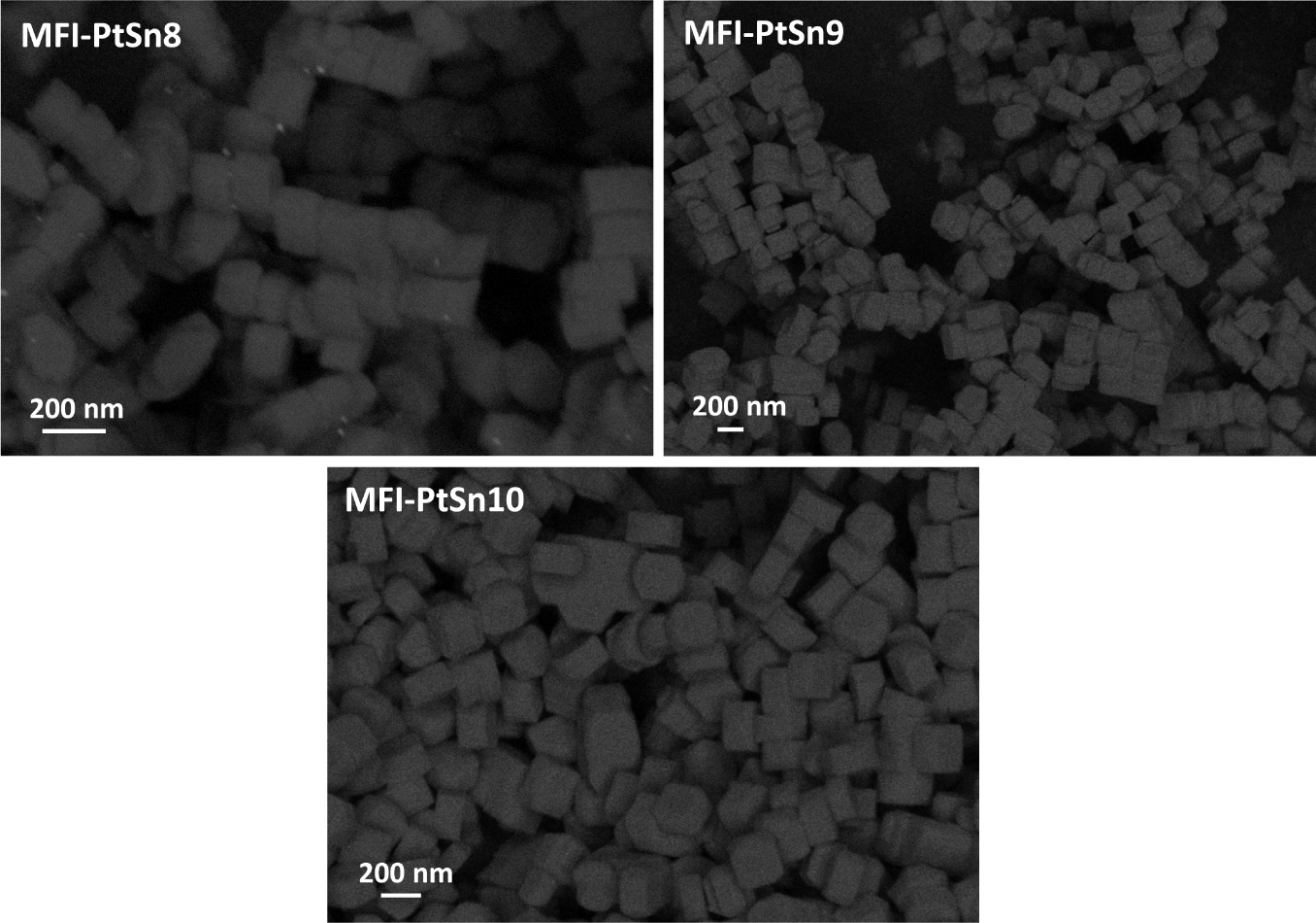
FESEM images of the calcined MFI-PtSn8, MFI-PtSn9, and MFI-PtSn10 samples.

**Figure 8:**
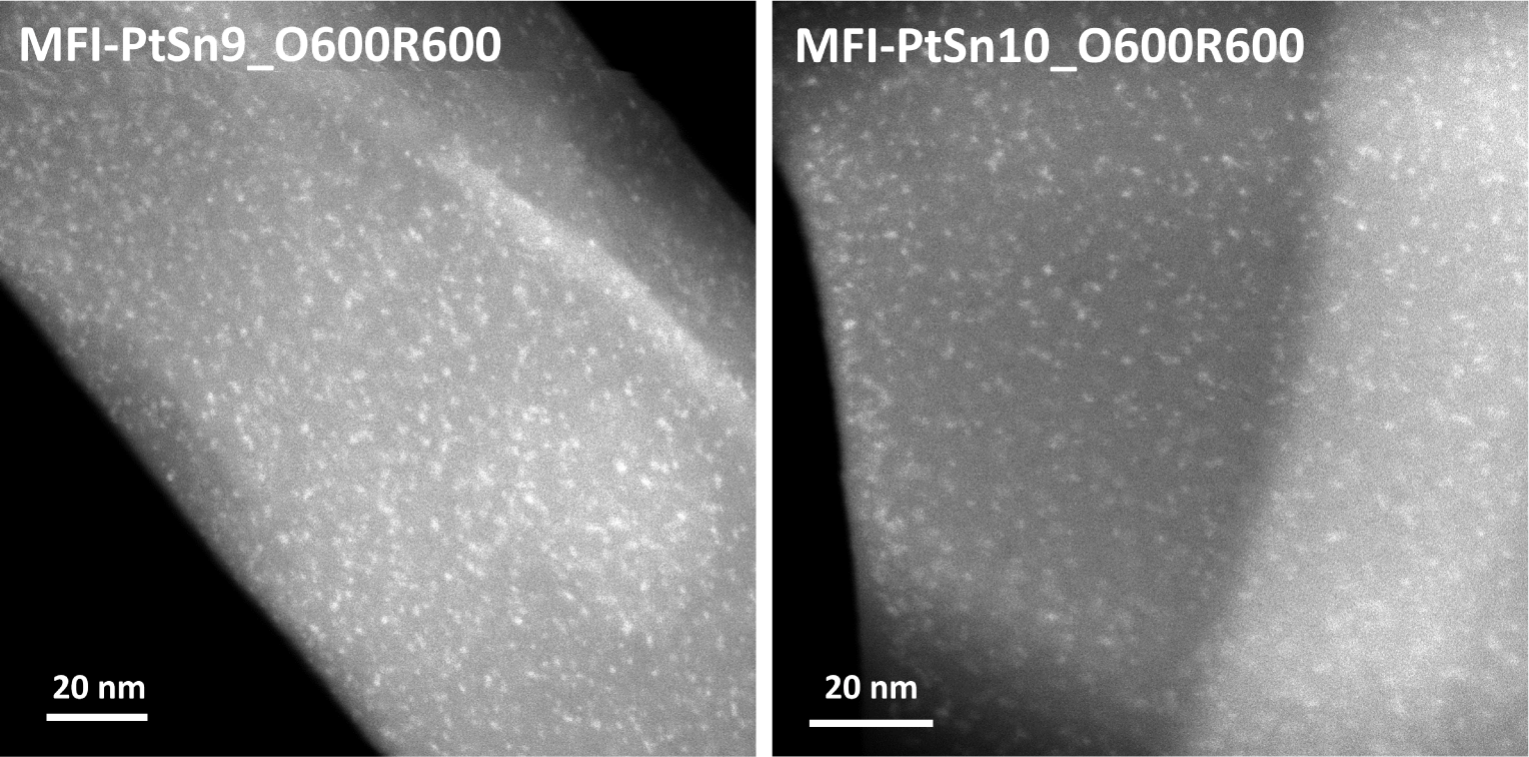
STEM images of the MFI-PtSn9 and MFI-PtSn10 samples after being subjected to calcination and reduction treatments at 600 °C in air and hydrogen, respectively (O600R600).

The PXRD patterns of MFI-PtSn8-10 show the formation of MFI as a pure phase (Figure 1), and the corresponding FESEM and STEM images confirm the virtual absence of amorphous tin-silicate particles (Figure 7). However, the sample with the highest Sn content, MFI-PtSn8, shows symptoms of metal aggregation after calcination, something that is not observed at lower Sn contents. Analysis of MFI-PtSn8 by STEM confirms the presence of significant amounts of Pt nanoparticles on the surface of the MFI crystals (Figure S9a), but the zeolite appears also to be full of very tiny metal clusters of lower Z-contrast, only observable at high magnifications (Figure S9b). Taking into account that the silanol/K-siloxy nests are inferred critical to stabilize ∼1 nm Pt clusters after multiple high-temperature reduction–oxidation–reduction cycles, one plausible explanation to the decreasing catalyst stability with increasing Sn contents, at K loadings below the maximum allowed K stoichiometry of ∼0.7 wt %, is that extra-framework Sn species outcompete Pt for occupying those positions (when Sn is in large excess). At lower Sn contents, in contrast, brighter clusters, well-dispersed throughout the MFI crystals, can be observed in the corresponding STEM images after one oxidation–reduction cycle at 600 °C (see MFI-PtSn9_O600R600 and MFI-PtSn10_O600R600 in Figure 8). This result is consistent with the observation that subnanometric Pt clusters in Sn-free Pt@K-MFI catalysts are highly stable under conditions that cause dramatic sintering of the metal inside common Al-containing MFI zeolites (e.g., after switching 50 times from pure H_2_ to air at a constant temperature of 600 °C).^21^

ICP analysis demonstrates that the Sn and K contents in the final solids agree with a ∼100% incorporation of the metals in the synthesis gels (Table 1). To test that the K and Sn fully incorporate into the zeolite crystals at these concentrations of K and Sn in the synthesis gels, we proceeded to wash the as-prepared samples with NaOH, as previously done, and confirmed that the content of K and Sn (as well as Pt) remains unaffected by the base-treatment. This result is a strong indication that tin-precipitates are avoided when the amount of K introduced in the synthesis gel is <0.7 wt %, even if the amount of Sn is high.

It is also worth noting that the overall density of structural defects in calcined PtSn@K-MFI samples that contain stoichiometric amounts of K is affected only moderately by the presence of Sn, as inferred by ^29^Si MAS NMR and FTIR spectroscopies (see Figure S10). For example, the ^29^Si MAS NMR band at −102 ppm, associated with Q_3_ defective sites, and the IR band at ∼3550 cm^–1^, associated with inner structural defects, are both only slightly lower in sample MFI-PtSn10, containing ∼0.5 wt % Sn, compared to sample MFI-PtSn9, containing ∼1 wt % Sn (see Figure S10).

At this point, we decided to evaluate the impact of the synthesis conditions and the resulting metal structures on the performance of K-PtSn@MFI catalysts for the PDH reaction.

### 2.4 PDH Catalytic Performance

Our results demonstrate that the distribution and uniformity of metal species in PtSn@K-MFI samples made via one-pot methods are very sensitive to changes in the K and Sn contents in the synthesis gels and that these two variables are closely interconnected due to competing amorphous phases that precipitate as impurities when K and Sn are in excess.

**Figure 9:**
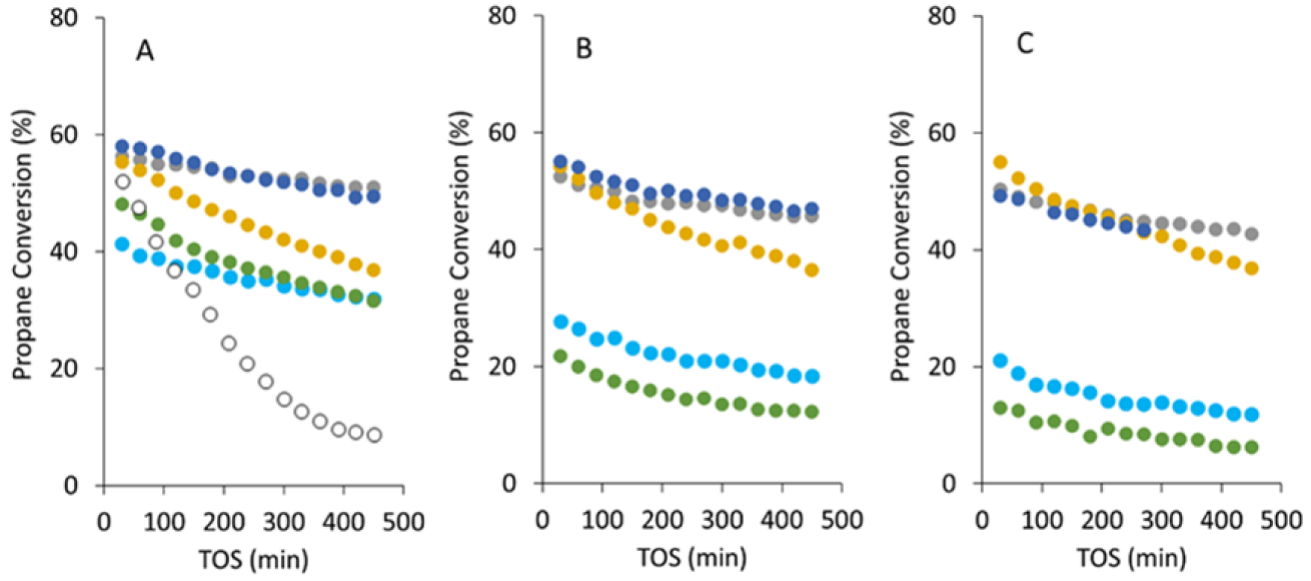
Propane conversion as a function of the time-on-stream for the direct propane dehydrogenation reaction using representative PtSn@K-MFI catalysts. A) Catalysts after a first calcination in air and reduction in H_2_ for 1 h at 600 °C. B) Catalysts after one consecutive regeneration in air at 600 °C for 1 h, followed by reactivation in H_2_ at 600 °C for 1 h. C) Catalysts after a second consecutive regeneration in air at 600 °C for 1 h, followed by reactivation in H_2_ at 600 °C for 1 h. Color of the symbols correspond to MFI-PtSn3 (gray); MFI-Pt6 (white); MFI-PtSn8 (light blue); MFI-PtSn9 (dark blue); MFI-PtSn10 (yellow); and PtSn/SiO_2_ (green). Catalyst compositions are indicated in Table 1. Reaction conditions: WHSV = 17.3 h^–1^ (on a propane basis); atmospheric pressure; 600 °C; 3:1 N_2_/propane molar ratio.

To correlate these structures with their catalytic performance in the PDH reaction, we tested catalysts MFI-PtSn3 (synthesized at high K and Sn loadings, thus containing significant amounts of tin-silicates), MFI-PtSn8 (stoichiometric K content and high Sn loading), MFI-PtSn9 (stoichiometric K content and intermediate Sn loading), and MFI-PtSn10 (stoichiometric K content and low Sn loading) after calcination and reduction in O_2_ and H_2_, respectively, at 600 °C for 1 h. The evolution of the propane conversion with time-on-stream is summarized in Figure 9 and includes data over three consecutive reaction/regeneration cycles at 600 °C in O_2_ and H_2_ (needed to remove coke and reactivate the catalyst). For comparison purposes, the performance of a Sn-free, Pt@K-MFI sample (MFI-Pt6) is also reported after one use, along with the performance of a PtSn/SiO_2_^22^ after three reaction/regeneration cycles. The selectivity of these catalysts exceeds, in all cases, 92% (see Figure S11), except that of the MFI-Pt6 sample, which stays 85–90% due to enhanced hydrogenolysis and coking when Sn is absent, consistent with the well-known role of this promoter on the selectivity of Pt.^25,26^ Accordingly, Figure 9 shows that time-on-stream deactivation is also the greatest with this catalyst. The selectivity of the best catalysts in this series, at high Sn contents, is >96%.

On the other hand, it is very clear that compared to other state-of-the-art PtSn catalysts, for example, recent PtSn/SiO_2_ catalysts,^22^ the optimized PtSn@K-MFI materials a) are substantially more active after the first H_2_ activation (∼58% conversion, very close to the theoretical equilibrium conversion under the selective reaction conditions, vs ∼40% with the reference PtSn/SiO_2_, both tested at a propane WHSV of 17.3 h^–1^); b) deactivate slower over time-on-stream (∼4 times slower); c) regenerate in O_2_ without significant deactivation (decay from ∼58% conversion to ∼50 −55%, depending on the Sn content, vs ∼48 to ∼13% in the case of PtSn/SiO_2_); and d) are more selective to propylene, particularly after O_2_ regeneration (∼96 vs <90%), as observed in Figures 9 and S11. STEM images of the spent PtSn/SiO_2_ catalyst demonstrate the transition from well-dispersed ∼2 nm PtSn nanoparticles, before reaction, to particles between 2 and 15 nm after reaction and regeneration (Figure S12). The bimodal particle size distribution is consistent with an Ostwald-ripening sintering mechanism typical of supports unable to stabilize atomically dispersed PtO_*x*_ species in O_2_ at moderate-to-high temperatures.^27^ Similar problems affect commercial PtSn/Al_2_O_3_ formulations, which must be subjected to additional oxychlorination processes to recuperate and reimpregnate Pt after coke burn operations, to mitigate activity losses caused by Pt sintering.^12,28^ The best PtSn@K-MFI catalysts developed here also outperformed the PtSn/SiO_2_ reference when tested in the PDH reaction at high concentrations of propane (Figure S13). More specifically, sample MFI-PtSn9 is able to attain equilibrium conversion (∼30% at 550 °C) at a WHSV of 300 h^–1^, whereas the PtSn/SiO_2_ reference stays at ∼15% conversion at a WHSV of 150 h^–1^; and after regeneration in air at 600 °C, the zeolite-supported sample maintains a clear conversion and selectivity advantage relative to the amorphous SiO_2_ counterpart (Figure S13). Nevertheless, we note that both TOS deactivation and stability upon regeneration appear to be more challenging when highly concentrated propane streams are reacted, as expected.

**Figure 10:**
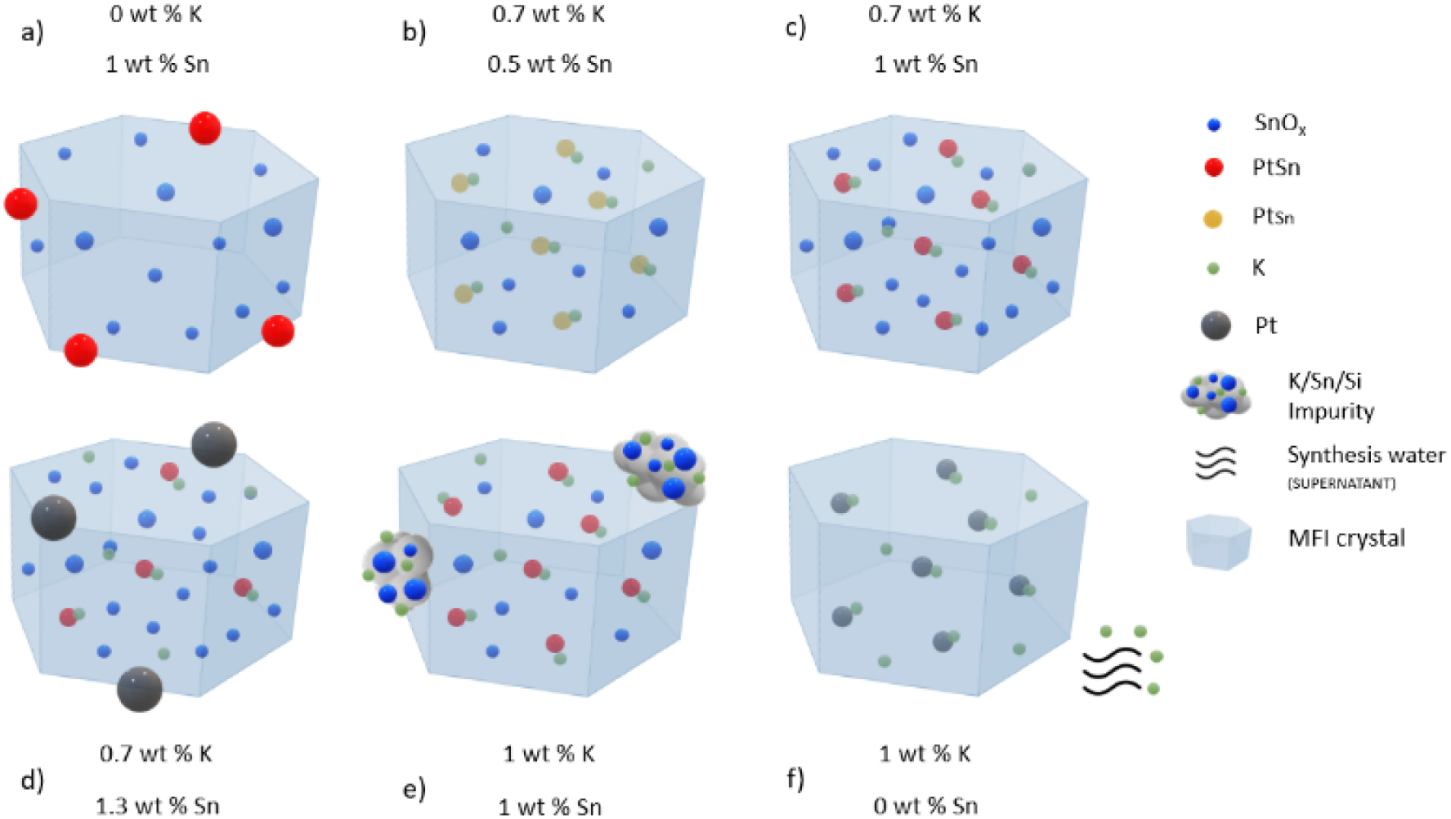
Summary of structures in calcined and reduced Pt-Sn@K-MFI materials synthesized by a one-pot methodology, as inferred in this work by EDX-STEM, FESEM, ICP analysis, EXAFS, NMR, and DFT calculations for synthesis gels with variable nominal compositions. The indicated K and Sn contents are the wt % of Sn and K, respectively, in the synthesis gels, on a SiO_2_ basis. The loading of Pt is 0.4 wt % in all cases. (a) The lack of K results in the formation of large PtSn outside the MFI crystals, which leads to low catalytic activity; b), c), and d) are structures synthesized with the maximum K that can be introduced into the zeolite (0.7 wt % K), at variable Sn content. Up to ∼ 1 wt % Sn, increasing the amount of Sn increases the number of Pt–Sn interactions (red species suggest more Sn-enrichment compared to yellow species). Beyond that point, part of the Pt escapes the zeolite crystals and grows as large Pt-rich particles. Samples at stoichiometric K amounts and moderate Sn deliver optimal activity, TOS deactivation, and regenerability; e) the excess of K above 0.7 wt % results in the precipitation of tin-silicates that are mere spectators in the PDH reaction; f) in the absence of Sn, the excess of K does not incorporate into the final solid and leaves the material during synthesis washing operations. In all these synthesis, K-silanol-siloxy nests play critical roles in the stabilization of subnanometric PtSn clusters located in the sinusoidal channels of MFI, near the K centers.

Because sample MFI-PtSn8 incorporates a non-negligible amount of Pt outside the MFI crystals (Figures 7 and S9), it is not surprising that this catalyst also deactivates relatively fast both during the PDH reaction (slope of the conversion vs time curve in Figure 9) and upon regeneration. Samples MFI-PtSn3 and MFI-PtSn9, in contrast, provide the slowest TOS deactivation in this series and a moderate loss of activity upon regeneration. These two samples, surprisingly, behave virtually identical in the PDH test, despite obvious differences in the composition and structures in the final solids (*vide supra*, and summarized in Figure 10). Thus, it is reasonable to speculate that both samples contain similar active sites, even when synthesized at different nominal gel compositions and even when the former incorporates impurities of K and Sn as large external tin-silicates, which appear to behave as mere spectators. Our data does not inform, however, if only one or otherwise various types of Sn species, finely dispersed inside the MFI crystals, are involved in the formation of Pt–Sn bonds during the H_2_ treatments.

Finally, the comparison between samples MFI-PtSn8, MFI-PtSn9, and MFI-PtSn10 is interesting because these three samples are synthesized with the correct stoichiometric amount of K in the synthesis gels, but at increasing Sn loadings. This situation largely avoids the formation of external tin-silicate precipitates and induces the vast majority of the Sn to penetrate into the zeolite pores, probably enhancing the Sn distribution/speciation within the resultant MFI crystals and maybe explaining the shortened reduction time observed. In this scenario, the higher the Sn content inside the zeolite, the greater the amount of Pt that we infer escapes from the zeolite channels after redox stress, corresponding to a loss of conversion after regeneration that follows the order MFI-PtSn8 (greatest loss) > MFI-PtSn9 > MFI-PtSn10 (comparison of the conversions at times = 0 in Figure 9). FESEM and STEM images of the spent catalysts show that the abundance of large (undesired) Pt ensembles also follows the same order, being minimal in MFI-PtSn10 (Figures S14 and S15), where the vast majority of the metal remains as subnanometric metal clusters after this strenuous test.

**Figure 11:**
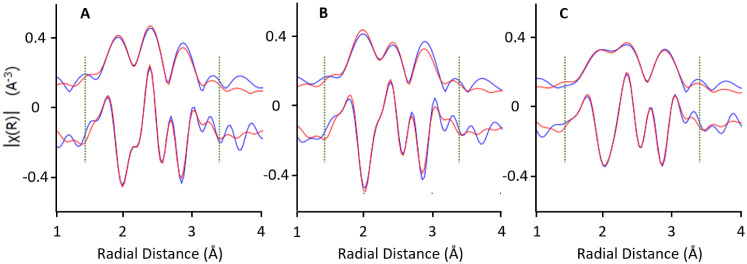
Magnitude and real parts of the Fourier transform EXAFS spectra (R-space without phase-correction) at k-weight = 2 for the MFI-PtSn9, and MFI-PtSn10 samples after reduction in H_2_ for 1 h at 600 °C (A and B plots, respectively), and for sample MFI-PtSn10 after reduction in H_2_ for 12 h at 600 °C (plot C). The green dotted lines indicate the fit window. All spectra were taken at 50 °C.

Sample MFI-PtSn10, the most stable upon regeneration, deactivates, however, faster over time-on-stream. To understand the different on-stream deactivation behavior of MFI-PtSn9 and MFI-PtSn10, both presenting negligible amounts of tin-silicate impurities and negligible amounts of Pt outside the MFI crystals, we characterized these samples by X-ray absorption spectroscopy (XAS) to unravel the nature of the PtSn interactions after their exposure to hydrogen at 600 °C for 1 h. Extended X-ray absorption fine structure (EXAFS) spectrum of the reduced PtSn@K-MFI samples (see Figure 11), along with quantitative fitting of the EXAFS parameters in Table 3, provides direct evidence of the Pt–Sn bonding, indicating that short reduction times in H_2_ (∼1 h) at 600 °C are sufficient to form the Pt–Sn bonds. The metal–metal bond distances are found within 2.71–2.74 Å for Pt–Pt and 2.55–2.62 for Pt–Sn. Interestingly, the Pt–Pt and Pt–Sn coordination numbers for the MFI-PtSn9 sample (1 wt % Sn) are 4.6 and 1.5, respectively, while those for MFI-PtSn10 (0.5 wt %) are 5.7 and 0.8, respectively, clearly indicating the formation of Pt-rich nanoparticles in both cases, more so when the Sn content is the lowest (MFI-PtSn10, see Table 3). The increased number of Pt–Sn bonds in sample MFI-PtSn9, containing a higher Sn content, correlates well with the less pronounced conversion versus time deactivation in Figure 9. This sample attains similar TOS deactivation to previous PtSn@K-MFI catalysts, without the requirement of long activation times in H_2_ prior to the reaction.^14^ In agreement with these results, dropping the Sn content below that of MFI-PtSn10, for example, by incorporating only 0.2 wt % Sn into the final solid (sample MFI-PtSn11), further aggravates the TOS deactivation problem (Figure S16). Moreover, in cases where the catalyst lacks sufficient PtSn bonds after a short H_2_ activation (i.e., typically, in catalysts that include relatively small amounts of mobile Sn species, such as in MFI-PtSn10), it is possible to promote the formation of new PtSn interactions by increasing the exposure to H_2_ (Table 3 and Figure 11C), which leads to decreased TOS deactivation (Figure S16B), as previously reported.^14^

**Table 3:** EXAFS Data at the Pt LIII Edge Characterizing the PtSn@K-MFI Samples after Being Reduced in H_2_ for 1 or 12 H at 600 °C^a^

Sample	Shell	N	*R* (Å)	Δσ^2^ (Å^2^)	Δ*E*_0_ (eV)
MFI-PtSn9 (1 h in H_2_)	Pt–Pt	4.6	2.74	0.01	4.9
	Pt–Sn	1.5	2.57	0.008	0.6
MFI-PtSn10 (1 h in H_2_)	Pt–Pt	5.7	2.74	0.011	4.3
	Pt–Sn	0.8	2.60	0.005	2.0
MFI-PtSn10 (12 h in H_2_)	Pt–Pt	4.2	2.74	0.009	5.4
	Pt–Sn	1.1	2.60	0.011	5.5
MFI-PtSn9 (PDH-spent)	Pt–Pt	6.8	2.75	0.012	7.5
	Pt–Sn	2.0	2.55	0.010	–4.3

a Notation: *N*, coordination number; *R*, distance between absorber and backscatterer atoms; Δσ^2^, Debye–Waller factor; Δ*E*_0_, inner potential correction. Error bounds (accuracies) characterizing the structural parameters obtained by EXAFS spectroscopy are estimated to be as follows: coordination number *N*, ∼ 20%; distance *R*, ∼ 0.02; Debye–Waller factor Δσ^2^, ∼ 20%; and inner potential correction Δ*E*_0_, ∼ 20%.

Finally, EXAFS was also used to evaluate the coordination of Pt in the MFI-PtSn9 sample after the PDH reaction. The spent MFI-PtSn9 catalyst shows a slight increase in the average Pt–Pt and Pt–Sn coordination numbers compared to the fresh counterpart, although the overall Pt–Pt to Pt–Sn molar ratio stays virtually unaffected (comparison between the first and last entries in Table 3).

Figure 10 summarizes the relationships between gel (nominal) composition, composition and structures in the final solids, and catalytic performance according to the present investigation. They reveal subtle, complex surface chemistries that are important to rationalize the characteristics of K/Sn/Pt catalysts synthesized by one-pot methodologies.

## 3 Conclusions

The K and Sn contents were systematically varied in the synthesis of the PtSn@K-MFI system to evaluate the metal dispersion and stability along the MFI crystallites. Experimental and theoretical calculations demonstrate that K is no longer incorporated in siliceous MFI crystals above ∼0.65 wt %, corresponding to a stoichiometry of ∼1 K per unit cell of MFI. Above this stoichiometry, K is not incorporated into the final solids, unless significant amounts of Sn are simultaneously present, leading to the formation of tin-silicate precipitates.

The performance of different optimized PtSn@K-MFI catalysts and standard reference materials was tested in the PDH reaction. Higher TOS deactivation catalytic profiles are observed with low Sn loadings (below 0.5 wt %), but these materials present excellent regenerability after consecutive PDH reactions. In contrast, Sn content close to 1 wt % seems optimal to minimize TOS deactivation in the PDH reaction due to the maximization of Pt–Sn bonds, as revealed by EXAFS, but consecutive regenerations after long-time PDH reaction cycles result in significant metal sintering. Increasing Sn contents within MFI crystallites facilitates Pt sintering and, thus, occurring catalyst deactivation upon regeneration cycles. The performance of optimized catalysts in this work is also markedly superior relative to well-established literature references, as PtSn/SiO_2_. The results obtained here clearly highlight the remarkable relevance that fine-tuning rationalizations of simple one-pot synthesis approaches can have on the final atomic and subnanometric metal interactions, as a result of complex interconnected nucleation/crystallization processes, and consequently, in the catalytic and sintering-resistance properties when exposed to highly demanding industrial conditions.

## Supporting Information Available

Description of experimental details and methods, microscopy studies (FESEM and STEM images) of different PtSn@K-MFI materials, optimized theoretical geometries of MFI frameworks containing TPA and/or K, product selectivities for the PDH reaction, as well as ^29^Si MAS NMR and FTIR spectra of the optimized PtSn@K-MFI materials (PDF)
