# Supplementary material for: Resolving Complex K–Pt–Sn Interactions in PtSn@K-MFI Catalysts for Alkane Dehydrogenation
Source: J Am Chem Soc. 2025 Apr 3;147(15):12833–44. doi: 10.1021/jacs.5c01536 (PMC12006991; doi:10.1021/jacs.5c01536)
Supplement: Supplementary file 1 — ja5c01536_si_001.pdf [file ja5c01536_si_001.pdf]

Supporting Information

Resolving complex K-Pt-Sn interactions in PtSn@K-MFI catalysts for alkane dehydrogenation

Adrián Martínez Gómez-Aldaraví,[1] Reisel Millán,[1] Isabel Millet,[1] Aroa Alós,[1] Alejandro Vidal-Moya,[1] Randall J. Meyer,[2] Cristina Martínez,[1] Avelino Corma[1], Mercedes Boronat,[1] Pedro Serna,[1]* Manuel Moliner[1]*

[1] Instituto de Tecnología Química, Universitat Politècnica de València-Consejo Superior de Investigaciones Científicas, Avenida de los Naranjos s/n, 46022 València, Spain

[2] ExxonMobil Technology and Engineering Co., Annandale, New Jersey 08801, United States

*Corresponding authors: E-mail addresses: psername@itq.upv.es; mmoliner@itq.upv.es

Experimental Section

S.1.- Zeolite synthesis

S.1.1.- Synthesis of MFI-PtSn1

First, 5.0 g of K-free tetrapropylammonium hydroxide (TPAOH) solution (40 wt.%, from Alfa-Aesar, product code: 17456.22) and 6.24 g of a K-containing TPAOH solution (20 wt.%, from Sigma-Aldrich) were mixed with 17.0 g of distilled water at room temperature. Then, 8.24 g Tetraethyl orthosilicate (TEOS) was hydrolyzed with the previous tetrapropylammonium hydroxide solution (TPAOH) at room temperature for 6 h under stirring. Afterwards, 0.36 g of a 20 wt.% aqueous solution of $SnCl_4 \cdot 5H_2O$ (Sigma-Aldrich, 98%) was added to the mixture and maintained under stirring for 5 min.

In parallel, a 0.52 g of a Pt-complex solution was prepared by mixing 0.25 g of a chloroplatinic acid solution (8 wt.%, H_2PtCl_6, Sigma-Aldrich) and 0.27 g of ethylenediamine (Sigma-Aldrich, 99%), and maintained under stirring for 20 min. Then, the Pt-complex solution was added to the previous mixture and maintained under rigorous stirring for additional 20 min. The final mixture was transferred to a Teflon-lined autoclave and heated in an oven at 175°C for 96 h under static conditions. The solid product was filtered and washed with distilled water and acetone and then dried at 60°C.

S.1.2.- Synthesis of MFI-PtSn2

Firstly, 8.12 g of K-free tetrapropylammonium hydroxide (TPAOH) solution (40 wt.%, from Alfa-Aesar, product code: 17456.22) was mixed with 13.93 g of distilled water and 6.15 g of an aqueous KCl solution (0.95 wt.%) at room temperature. Then, 8.24 g Tetraethyl orthosilicate (TEOS) was hydrolyzed with the previous tetrapropylammonium hydroxide solution (TPAOH) at room temperature for 6 h under stirring. Afterwards, 0.50 g of a 20 wt.% aqueous solution of $SnCl_4 \cdot 5H_2O$ (Sigma-Aldrich, 98%) was added to the mixture and maintained under stirring for 5 min.

In parallel, a 0.52 g of a Pt-complex solution was prepared by mixing 0.25 g of a chloroplatinic acid solution (8 wt.%, H_2PtCl_6, Sigma-Aldrich) and 0.27 g of ethylenediamine (Sigma-Aldrich, 99%), and maintained under stirring for 20 min. Then, the Pt-complex solution was added to the previous mixture and maintained under rigorous stirring for additional 20 min. The final mixture was transferred to a Teflon-lined autoclave and heated in an oven at 175°C for 96 h under static conditions. The solid product was filtered and washed with distilled water and acetone and then dried at 60°C.

S.1.3.- Synthesis of MFI-PtSn3

The MFI-PtSn3 sample was prepared by a similar one-pot synthesis approach as MFI-PtSn2, but modifying the amount of the $SnCl_4$ solution. In this case, 0.36 g of a 20 wt.% aqueous solution of $SnCl_4{\cdot}5H_2O$ (Sigma-Aldrich, 98%) was added to the mixture.

S.1.4.- Synthesis of MFI-PtSn4

The MFI-PtSn4 sample was prepared by a similar one-pot synthesis approach as MFI-PtSn2, but modifying the amount of the $SnCl_4$ solution. In this case, 0.25 g of a 20 wt.% aqueous solution of $SnCl_4{\cdot}5H_2O$ (Sigma-Aldrich, 98%) was added to the mixture.

S.1.5.- Synthesis of MFI-PtSn5

The MFI-PtSn5 sample was prepared by a similar one-pot synthesis approach as MFI-PtSn2, but modifying the amount of the $SnCl_4$ solution. In this case, 0.18 g of a 20 wt.% aqueous solution of $SnCl_4{\cdot}5H_2O$ (Sigma-Aldrich, 98%) was added to the mixture.

S.1.6.- Synthesis of MFI-Pt6

The MFI-Pt6 sample was prepared by a similar one-pot synthesis approach as MFI-PtSn2, but without introducing the $SnCl_4$ solution in the preparation.

S.1.7.- Syntheses of MFI-7a and MFI-7b

The MFI-7a and MFI-7b samples were prepared by a similar one-pot synthesis approach as MFI-PtSn2, but without introducing the $SnCl_4$ and the Pt-ethylenediamine complex

solutions in the preparation for MFI-7a, and without introducing the $SnCl_4$, the KCl and the Pt-ethylenediamine complex solutions in the preparation for MFI-7b.

S.1.8.- Synthesis of MFI-PtSn8

The MFI-PtSn8 sample was prepared by a similar one-pot synthesis approach as MFI-PtSn2, but modifying the amount of the KCl and $SnCl_4$ solutions. In this case, 8.12 g of K-free tetrapropylammonium hydroxide (TPAOH) solution (40 wt.%, from Alfa-Aesar, product code: 17456.22) was mixed with 17 g of distilled water and 3.08 g of an aqueous KCl solution (0.946 wt.%) at room temperature. Then, 8.24 g Tetraethyl orthosilicate (TEOS) was hydrolyzed with the previous tetrapropylammonium hydroxide solution (TPAOH) at room temperature for 6 h under stirring. Afterwards, 0.47 g of a 20 wt.% aqueous solution of $SnCl_4{\cdot}5H_2O$ (Sigma-Aldrich, 98%) was added to the mixture and maintained under stirring for 5 min.

The Pt-complex preparation/addition as well as the mixture transfer to the autoclave and crystallization conditions were the same as described for the MFI-PtSn2 experiment.

S.1.9.- Synthesis of MFI-PtSn9

The MFI-PtSn9 sample was prepared by a similar one-pot synthesis approach as MFI-PtSn2, but modifying the amount of the KCl and $SnCl_4$ solutions. In this case, 8.12 g of K-free tetrapropylammonium hydroxide (TPAOH) solution (40 wt.%, from Alfa-Aesar, product code: 17456.22) was mixed with 17 g of distilled water and 3.08 g of an aqueous KCl solution (0.946 wt.%) at room temperature. Then, 8.24 g Tetraethyl orthosilicate (TEOS) was hydrolyzed with the previous tetrapropylammonium hydroxide solution (TPAOH) at room temperature for 6 h under stirring. Afterwards, 0.36 g of a 20 wt.% aqueous solution of $SnCl_4{\cdot}5H_2O$ (Sigma-Aldrich, 98%) was added to the mixture and maintained under stirring for 5 min.

The Pt-complex preparation/addition as well as the mixture transfer to the autoclave and crystallization conditions were the same as described for the MFI-PtSn2 experiment.

S.1.10.- Synthesis of MFI-PtSn10

The MFI-PtSn10 sample was prepared by a similar one-pot synthesis approach as MFI-PtSn2, but modifying the amount of the KCl and $SnCl_4$ solutions. In this case, 8.12 g of K-free tetrapropylammonium hydroxide (TPAOH) solution (40 wt.%, from Alfa-Aesar, product code: 17456.22) was mixed with 17 g of distilled water and 3.08 g of an aqueous KCl solution (0.946 wt.%) at room temperature. Then, 8.24 g Tetraethyl orthosilicate (TEOS) was hydrolyzed with the previous tetrapropylammonium hydroxide solution (TPAOH) at room temperature for 6 h under stirring. Afterwards, 0.18 g of a 20 wt.% aqueous solution of $SnCl_4 \cdot 5H_2O$ (Sigma-Aldrich, 98%) was added to the mixture and maintained under stirring for 5 min.

The Pt-complex preparation/addition as well as the mixture transfer to the autoclave and crystallization conditions were the same as described for the MFI-PtSn2 experiment.

S.1.11.- Synthesis of MFI-PtSn11

The MFI-PtSn11 sample was prepared by a similar one-pot synthesis approach as MFI-PtSn2, but modifying the amount of the KCl and $SnCl_4$ solutions. In this case, 8.12 g of K-free tetrapropylammonium hydroxide (TPAOH) solution (40 wt.%, from Alfa-Aesar, product code: 17456.22) was mixed with 17 g of distilled water and 3.08 g of an aqueous KCl solution (0.946 wt.%) at room temperature. Then, 8.24 g Tetraethyl orthosilicate (TEOS) was hydrolyzed with the previous tetrapropylammonium hydroxide solution (TPAOH) at room temperature for 6 h under stirring. Afterwards, 0.072 g of a 20 wt.% aqueous solution of $SnCl_4 \cdot 5H_2O$ (Sigma-Aldrich, 98%) was added to the mixture and maintained under stirring for 5 min.

The Pt-complex preparation/addition as well as the mixture transfer to the autoclave and crystallization conditions were the same as described for the MFI-PtSn2 experiment.

S.1.12.- Post-synthetic NaOH treatments

The as-synthesized MFI-PtSn2 sample was treated with a 20 wt.% NaOH aqueous solution (liquid/solid ratio of ~10) at 60°C, maintaining the mixture under stirring for 1 h. The solid product was filtered and washed with distilled water and acetone and then dried at 60°C.

S.1.13.- Calcination and reduction treatments

The as-synthesized samples were calcined in a tubular furnace under flowing air (75 ml/min) with a heating rate of 2°C/min until 600°C and maintained for 4 h. The samples were collected and reduced in flowing H_2 (75 ml/min), with a heating rate of 10°C/min, at 600°C and maintained for 2 h.

S.1.14.- Synthesis of PtSn/SiO_2 as comparative catalyst

The synthesis of the PtSn/SiO_2 as comparative catalyst has been prepared according to the methodology described in the literature.[1] In particular, of 0.52 g of a chloroplatinic acid solution (8 wt.%, H_2PtCl_6, Sigma-Aldrich) and 16 mg of stannous chloride (Sigma-Aldrich) were added to 0.8 ml of 0.1 M HCl solution. 5 gram of silica (Sigma-Aldrich, pore size 60 Å) was impregnated using the previous solution by incipient wetness impregnation. The final catalyst was dried overnight at 80°C. ICP analysis reveals a final Pt and Sn content of ~0.4 and ~0.23, respectively.

S.2.- Characterization

Powder X-Ray diffraction (PXRD) measurements were performed employing a multisample holder Philips X'Pert diffractometer fitted with a graphite monochromator, running at 35 mA and 40 kV and a Cu Kα radiation of 0.1542 nm of wavelength.

The chemical composition of the materials was determined by Inductively Coupled Plasma Optimal Emission Spectroscopy (ICP-OES) using a Thermo-Scientific iCAP PRO spectrometer after dissolving the solid samples in an acid mixture of HNO_3: HCl: HF (1:3:1 volumetric ratio).

The morphologies and crystal sizes of the different zeolite particles as well as the presence/absence of particles on the external surface of the zeolite crystals were studied by High Resolution Field Emission Scanning Electron Microscopy (HR-FESEM) employing a ZEISS GeminiSEM 500 microscope (ZEISS- Oxford Instruments) fitted with a Backscattered detector.

The presence of metal clusters within zeolite crystals was studied by High-angle annular dark-field scanning transmission electron microscopy (HAADF-STEM) employing a JEOL JEM-2100F (JEOL-Oxford Instruments) running at 200 kV.

MAS NMR spectra were recorded in a Bruker AVIII HD 400. Prior to record the ^{1}H MAS NMR and ^{1}H DQ-SQ MAS NMR spectra the samples were evacuated in vacuum at 150°C to remove the adsorbed water on the zeolite surface. The spectra were recorded using a Bruker 3.2 mm probe by spinning the sample at 20KHz. BaBa dipolar recoupling sequence was used to excite and reconvert double-quantum (DQ) coherences using an excitation time of 100 us (2 periods). ^{29}Si MAS NMR spectra were recorded at spinning rate of 5 kHz at 79.459 MHz with a 55° pulse length of 3.5 µs and a repetition time of 180 s. ^{29}Si chemical shift was referenced to tetramethylsilane.

Infrared spectra were measured with a Nicolet 710 FT IR spectrometer were made on self-supported wafers (10 mg.cm^{-1}) of original samples previously activated at 400°C and 10^{-2} Pa for 2 hours. After wafer activation, the base spectrum was recorded. The spectra were scaled according to the sample weight.

XAS experiments were performed at the 8-ID ISS beamline at National Synchrotron Light Source-II at Brookhaven National Lab. All spectra were collected in fluorescence mode using a 4-element solid state detector with reference foils used for edge alignment. Data was typically acquired ~200 eV before the edge jump and up to ~950 eV post-edge. Prior to data acquisition, samples were ground and ~ 4 mg was loaded into a 1.5 mm OD x 1.3 mm ID quartz capillary with quartz wool plugs to hold the sample in place. Gas flows were typically in the range of 5-10 sccm. The sample was radiatively heated by two resistive heaters placed ~1 mm from the sample. Typical heating ramps were in the range of 10°C/min. Typical scans

took ~ 2 min. Typical gas concentrations were: 10 sccm of 5 % O_2 in He for oxidation treatments, and 10 sccm of pure H_2 for reduction treatments. The experimental data was analyzed using the Demeter software package.[2,3] After appropriate background subtraction, data ranges were assessed based on the quality of data generally between k = 3-12 $Å^{-1}$ and for R= 1.2-3.4 Å. The amplitude factor for Pt was determined to be 0.83 from a Pt foil spectra, setting the coordination number to a fixed value of 12 to reflect the fcc structure. The Debye Waller factors, E_0 and bond distances for all bonds were optimized while employing a simultaneous fit of the k, k^2 and k^3 weighted Fourier transforms.

S.3.- Theoretical calculations

Methods. Periodic density functional theory (DFT) calculations were performed using the Perdew−Burke−Ernzerhof (PBE) exchange-correlation functional within the generalised gradient approach (GGA),[4,5] as implemented in the Vienna Ab-initio Simulation Package (VASP 5.4) code.[6] The valence density was expanded in a plane wave basis set with a kinetic energy cutoff of 400 eV, and the effect of the core electrons in the valence density was taken into account by means of the projected augmented wave (PAW) formalism.[7] Integration in the reciprocal space was carried out at the Γ-point of the Brillouin zone. Dispersion corrections to the energies were evaluated using the D3 Grimme's method,[8–10] with the Becke-Johnson damping,[11] as implemented in VASP. Electronic energies were converged to 10^{-5} eV and geometries were optimized until forces on atoms were less than 0.05 eV/Å. During geometry optimizations, the positions of all atoms in the system were allowed to relax without any restriction while keeping the unit cell parameters constant.

Models. The pure silica MFI structure was modelled by means of an orthorhombic unit cell containing 96 Si atoms and 192 O atoms ($Si_{96}O_{192}$), with lattice parameters a = 20.090, b = 19.738 Å, c = 13.142 Å, α = β = γ = 90° that were kept constant in all the models employed. From this system, five MFI-TPA models with $Si_{92}O_{192}N_4C_{48}H_{124}$ composition were generated that contain four TPA cations placed in the four channels intersections present in each unit cell and with the four associated silanol/siloxy nests distributed in different ways (Table S1).

Taking as starting point the most stable MFI-TPA model (MFI-TPA-D1 in Table S1), one K^+ cation and its corresponding OH^- counter-ion were introduced in different locations of the unit cell, including the cages and the straight and sinusoidal 10R channels, generating twelve MFI-TPA-K systems with $Si_{92}O_{193}N_4C_{48}H_{125}K$ composition. For each of these MFI-TPA-K models, a reference system containing the four silanol nest defects associated to TPA, one K^+ cation and its associated OH^- counter-ion in the same initial location was also optimized. These reference systems are labelled MFI-D1-K and have $Si_{92}O_{193}H_{17}K$ composition.

The energy of incorporation of a K cation (E_{inc}) was calculated as:

$$E_{inc} = E(\text{MFI-4TPA-K}) + E(\text{MFI-D1}) - E(\text{MFI-4TPA}) - E(\text{MFI-D1-K})$$

Where E(MFI-D1) is the total energy of the zeolite framework containing four silanol nests, E(MFI-4TPA) is the total energy of the zeolite framework containing four TPA cations with the four associated silanol/siloxy nests, E(MFI-4TPA-K) is the total energy of the MFI-4TPA system with an additional K cation and its associated defect, and E(MFI-D1-K) is the total energy of the reference system containing the four silanol nests, one K cation and its associated OH group in the same location as in MFI-4TPA-K.

The energy of incorporation of a second K was calculated as:

$$E_{inc} = E(\text{MFI-4TPA-2K}) + E(\text{MFI-4TPA}) - E(\text{MFI-4TPA-K}) - E(\text{MFI-4TPA-K})$$

Where E(MFI-4TPA-2K) is the total energy of a system containing four TPA cations with the four associated silanol/siloxy nets and two K cations with the two corresponding associated defects, with $Si_{92}O_{194}N_4C_{48}H_{126}K_2$ composition.

S.4.- Catalytic tests

Direct propane dehydrogenation (PDH) using diluted propane streams: The direct PDH reaction was performed in a fix-bed reactor under atmospheric pressure using propane/N_2 (32/10) as feed gas at 600°C and the products were analyzed by gas chromatography (GC). 50 mg of the calcined and pelletized catalyst (pellets ~0.2-0.4 mm) was diluted in 0.4 g of silicon carbide (pellets ~0.6-0.8 mm). Then, the catalyst was first calcined in air (75 ml/min)

at 600°C for 3 h with a ramp rate of 10°C/min from room temperature up to 600°C, followed by a purge in N_2 flow (75 ml/min) for 30 min and, finally, reduced by H_2 flow (35 ml/min) at 600°C for 1 h. After the reduction pre-treatment, the reactor was purged with an inert atmosphere (N_2, 75 ml/min) for 30 min and, then, the atmosphere was changed to reaction feed gas (10 mL/min of C_3 and 32 mL/min of N_2 as balanced gas) and the reaction was evaluated for 8 h. After the propane dehydrogenation reaction cycle, the catalyst was in-situ regenerated by calcination in air at 600°C for 2 h, followed by a reduction treatment in H_2 at 600°C for 1 h. Then, a second and a third catalytic cycle for the direct propane dehydrogenation was carried out using different C_3 WHSVs as in the first catalytic cycle.

Propane dehydrogenation (PDH) using highly-concentrated propane streams: The direct PDH reaction was performed in a fix-bed reactor (7 mm diameter) under atmospheric pressure using propane/N_2 (90/10) as feed gas at 550°C and the products were analyzed by gas chromatography (GC). 40 or 20 mg of the calcined and pelletized (pellets ~0.2-0.4 mm) PtSn/SiO_2 or MFI-PtSn9 catalysts, respectively, was diluted in 1.2 g of silicon carbide (pellets ~0.6-0.8 mm). Propane and N_2 were fed in separate lines with a molar ratio of 9:1 controlled by flowmeter and mixed in a preheater loaded with silicon carbide at 120°C. When the catalyst was loaded, the activation started by heating in air (50 ml/min) up to 600°C with a heating rate of 10°C/min. After reaching 600°C, the system was purged with N_2 (50 ml/min) for 30 min, followed by a reduction treatment in H_2 (50 ml/min) at 600°C for 1 h. When the activation is finished, the system is purged with N_2 (50 ml/min) for 30 minutes while cooling the temperature of the reactor to 550°C. Then, 52 ml/min C_3H_8 and 5.8 ml/min N_2 were fed into the bypass line and the gas composition was analyzed on-line by a Varian GC 3800 equipped with a Rt-Alumina BOND/Na_2SO4 column and two TCD detectors and one FID detector. When the gas composition was stable, the feeding gas was switched to reactor and the time was set as Time-On-Stream = 0 min. The reaction products were analyzed on-line with the same GC and method.

When one catalytic test was finished, the feeding gas mixture was stopped and the reactor was purged with N_2 for 30 min while heating to 600°C at a heating rate of 10°C/min. Then

the gas was switched to air (50 ml/min) and maintained for 3 hours to burn coke deposits. After coke removal, the system was purged with N_2 (50 ml/min) for 30 minutes to remove air. Then pure H_2 (50 ml/min) was fed into the reactor for activation of the catalyst, maintaining the reactor at 600°C for 1 h. When the activation is finished, the system is purged by N_2 (50 ml/min) for 15 minutes while cooling the temperature of the reactor down to 550°C and the catalyst was ready for the reuse test.

Supplementary Figures

Figure S1: FESEM images of the MFI-PtSn1 sample prepared using two different commercially-available TPAOH solution batches containing traces of K.

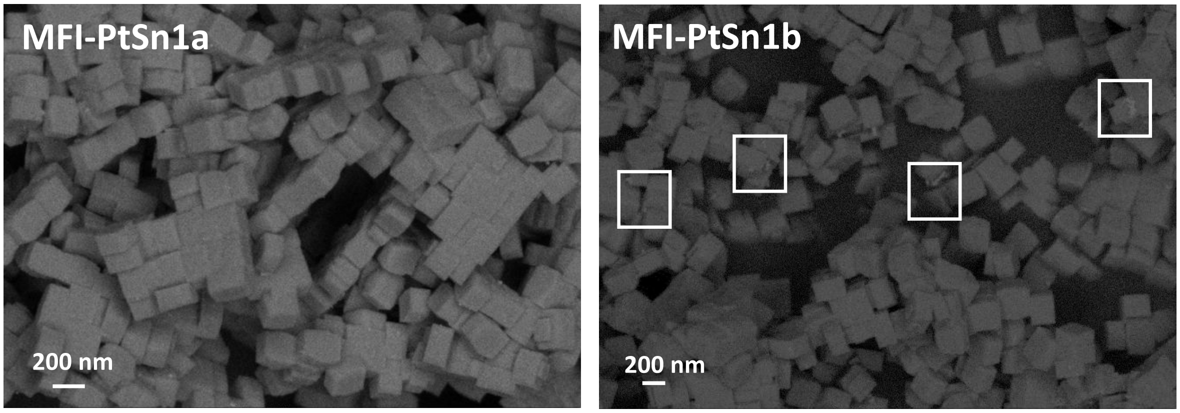

Figure S2: STEM images (a,b) and EDX/STEM analysis (c) of the MFI-PtSn1b sample after being subjected to calcination and reduction treatments at 600°C in air and hydrogen, respectively.

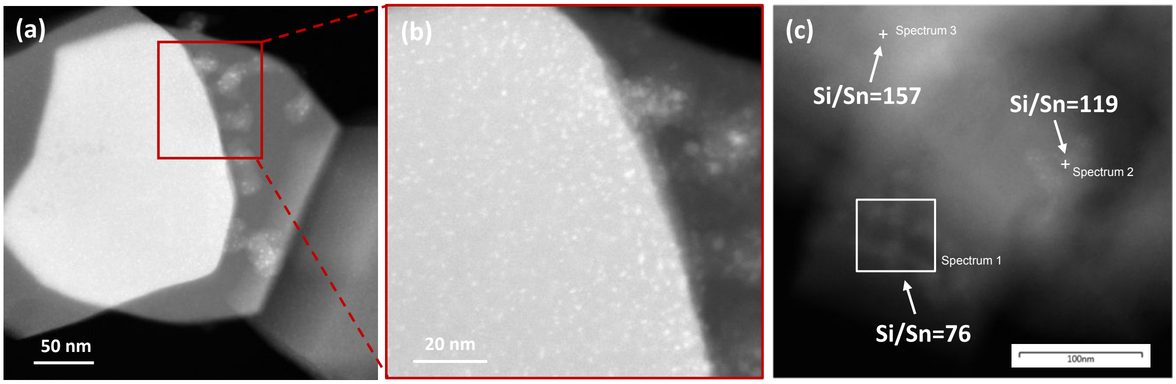

Figure S3: Amount of K (wt.%) incorporated in the final MFI-PtSn2-5 samples as the Sn content was decreased in the 1.4 to 0.5 wt.% range.

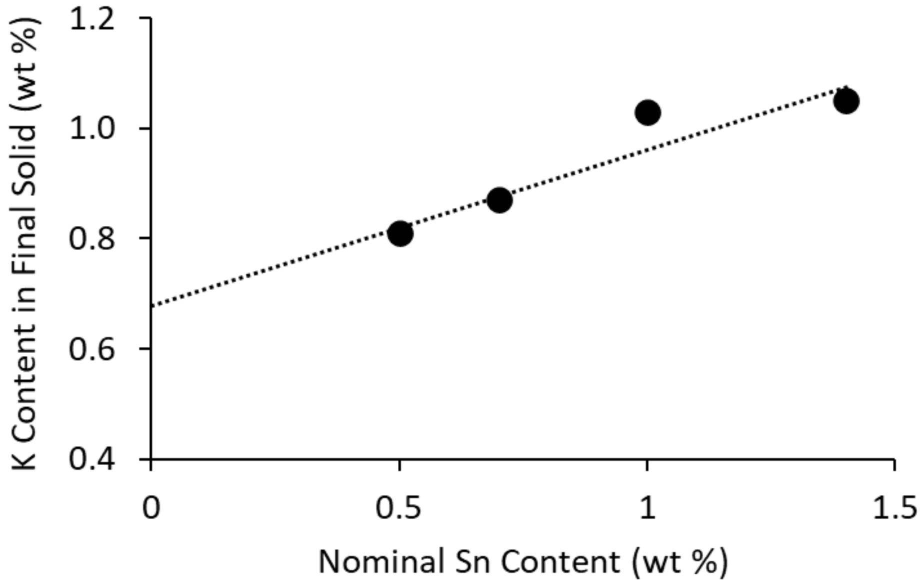

Figure S4: FESEM images of the as-prepared MFI-PtSn2 sample and after being subjected to a post-synthetic treatment with NaOH (20 wt.% aqueous solution at 60°C for 1 h, liquid/solid ratio ~10).

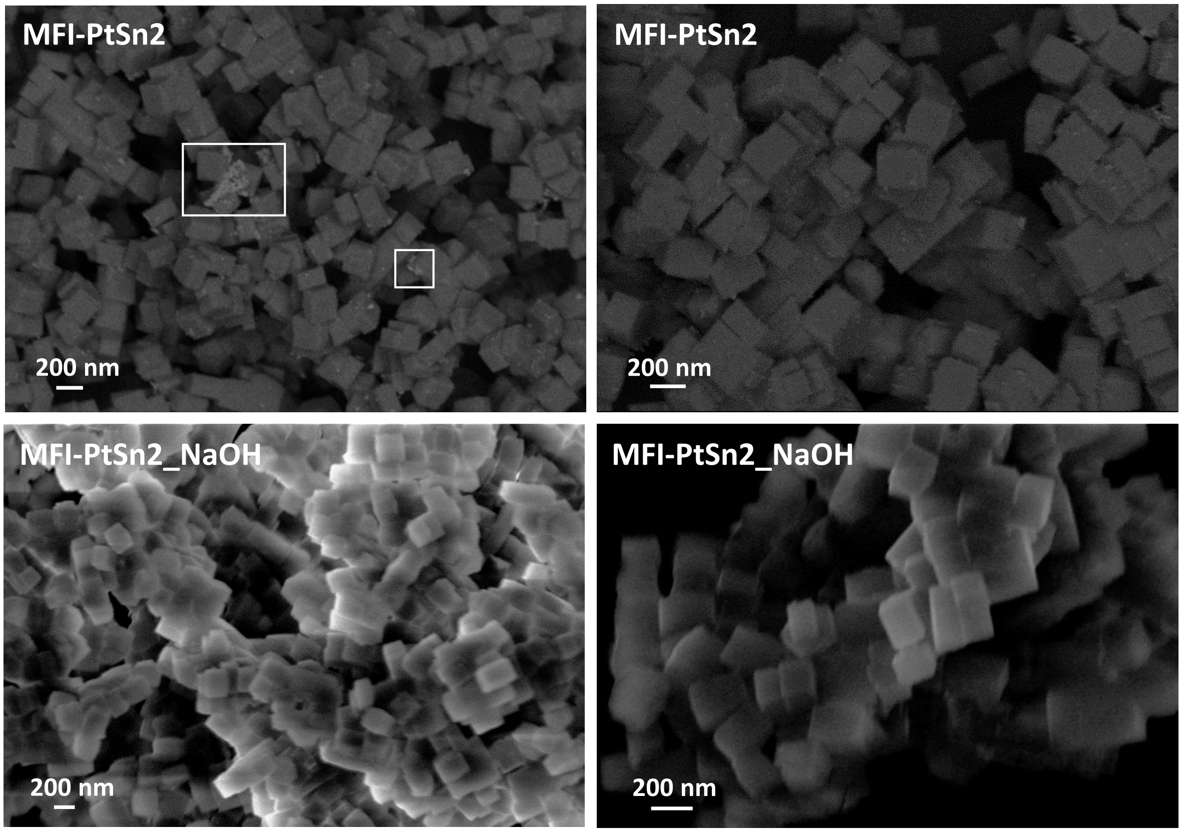

Figure S5: FESEM images of the Sn-free blank experiments MFI-Pt6 (K- and Pt-containing MFI), MFI-7a (K-containing and Pt-free MFI) and MFI-7b (K- and Pt-free MFI) materials.

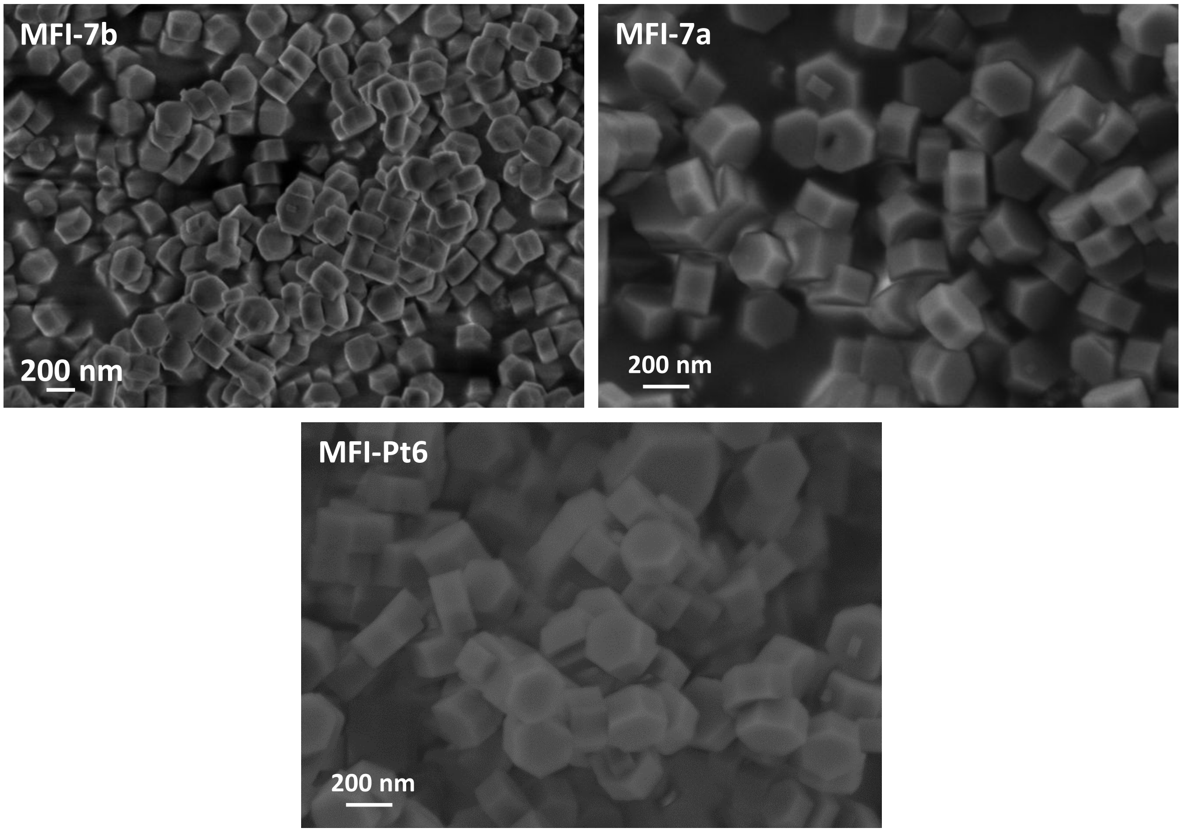

Figure S6: (a) Nest that results from an absent T site to produce three surrounding silanols (Si–OH) and one siloxy (Si–O⁻). (b) Nest produced by breaking at least one siloxane (Si–O–Si) linkage to create one siloxy and one silanol. M⁺ can be TPA⁺ or K⁺ in the as-prepared K-MFI system. Reproduced from ref. [12]

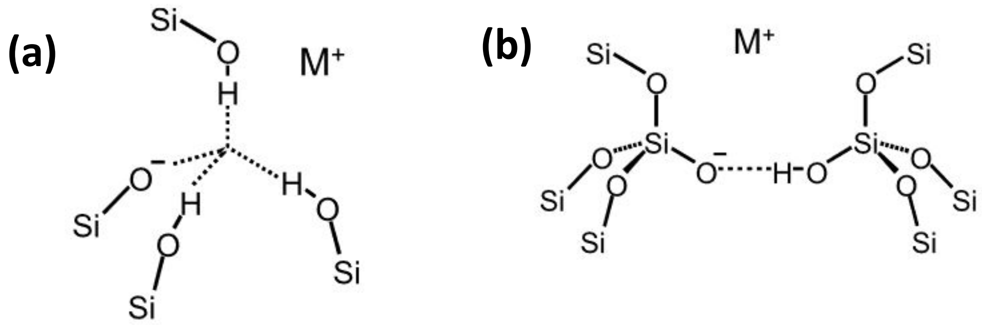

Figure S7: ^{1}H MAS NMR of different as-prepared MFI materials.

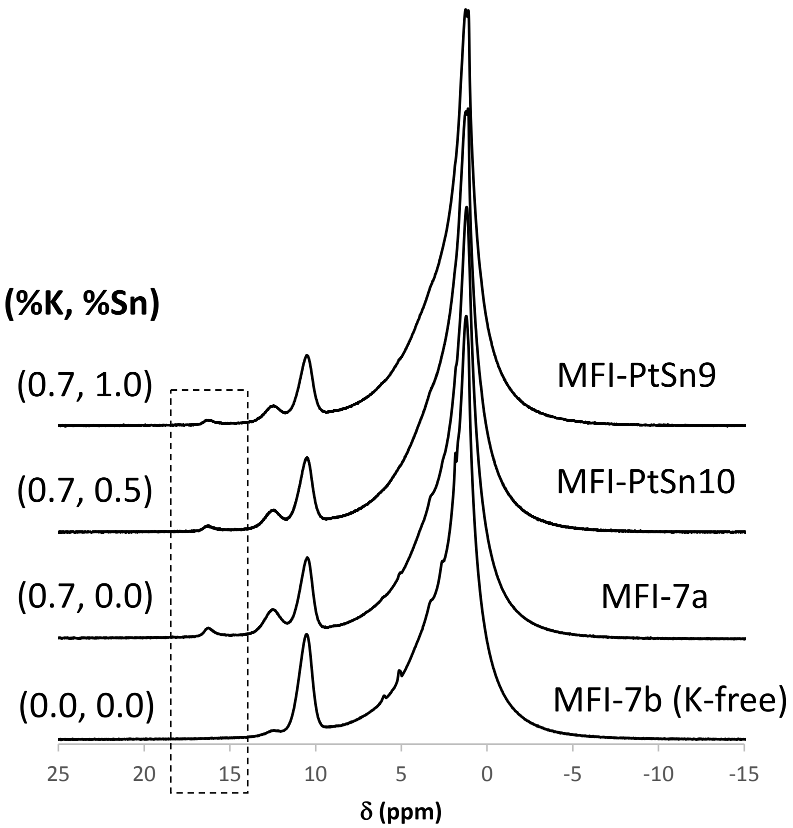

Figure S8: Optimized geometries of MFI-TPA, MFI-TPA-K and MFI-TPA-2K models. (a) View from the [100] direction showing the sinusoidal 10-ring channels and (b) view from the [010] direction showing the straight 10-ring channels. Framework Si and O atoms depicted as yellow and red sticks, N, C, H and K atoms depicted as blue, grey, white and pink balls.

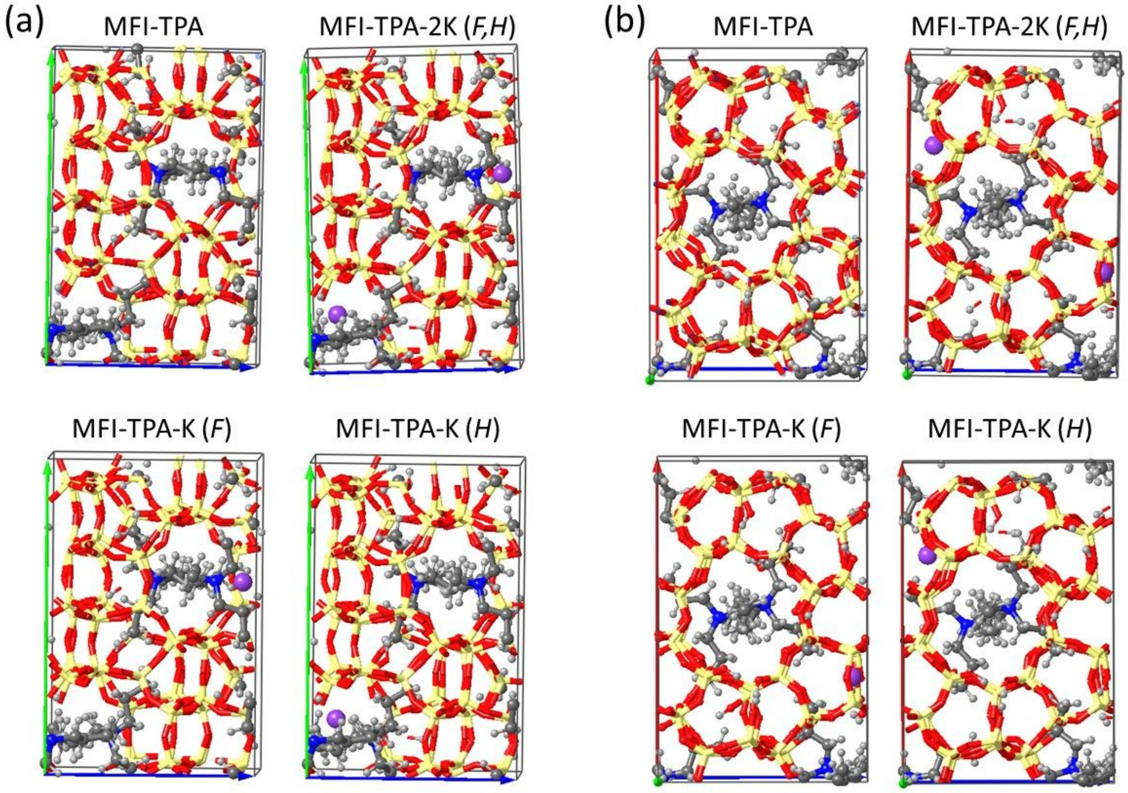

Figure S9. STEM images of the MFI-PtSn8 sample after being subjected to calcination in air and hydrogen at 600°C.

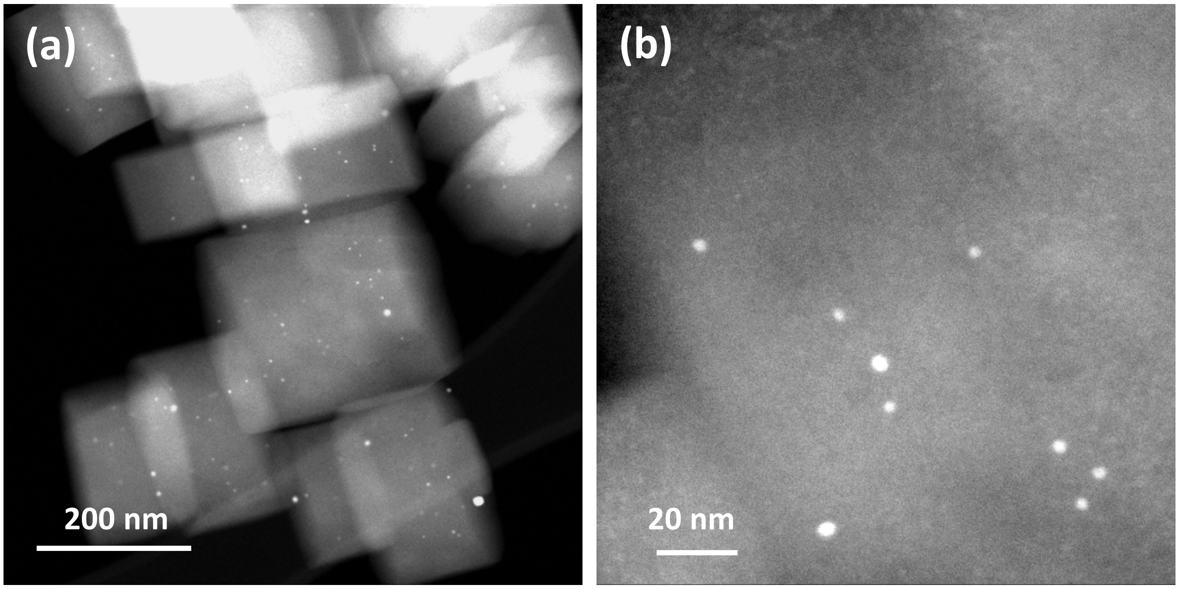

Figure S10: ^{29}Si MAS NMR (a) and FTIR spectra in the OH-region (3800-3400 cm^{-1}) (b) of the two MFI-PtSn9 and MFI-PtSn10 materials in their calcined forms.

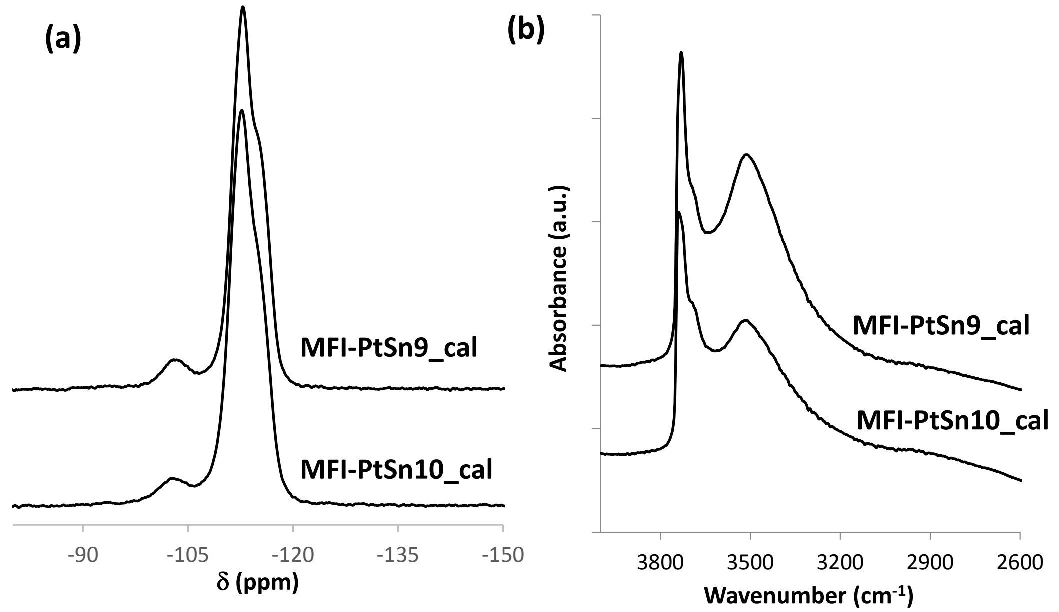

Figure S11. Propylene selectivity as a function of the time-on-stream for the direct propane dehydrogenation reaction using representative PtSn@K-MFI catalysts. A) Catalysts after a first calcination in air and reduction in H_2 for 1h at 600°C. B) Catalysts after one consecutive regeneration in air at 600°C for 1 h, followed by re-activation in H_2 at 600°C for 1h; C) Catalysts after a second consecutive regeneration in air at 600°C for 1 h, followed by re-activation in H_2 at 600°C for 1h. Colour of the symbols correspond to: MFI-PtSn3 (grey); MFI-Pt6 (white); MFI-PtSn8 (light blue); MFI-PtSn9 (dark blue); MFI-PtSn10 (yellow) and PtSn/SiO_2 synthesized according to [1] (green). Catalyst compositions indicated in Table 1. Reaction conditions: WHSV = 17.3 h^{-1} (on a propane basis); atmospheric pressure; 600°C; 3:1 N_2/propane molar ratio.

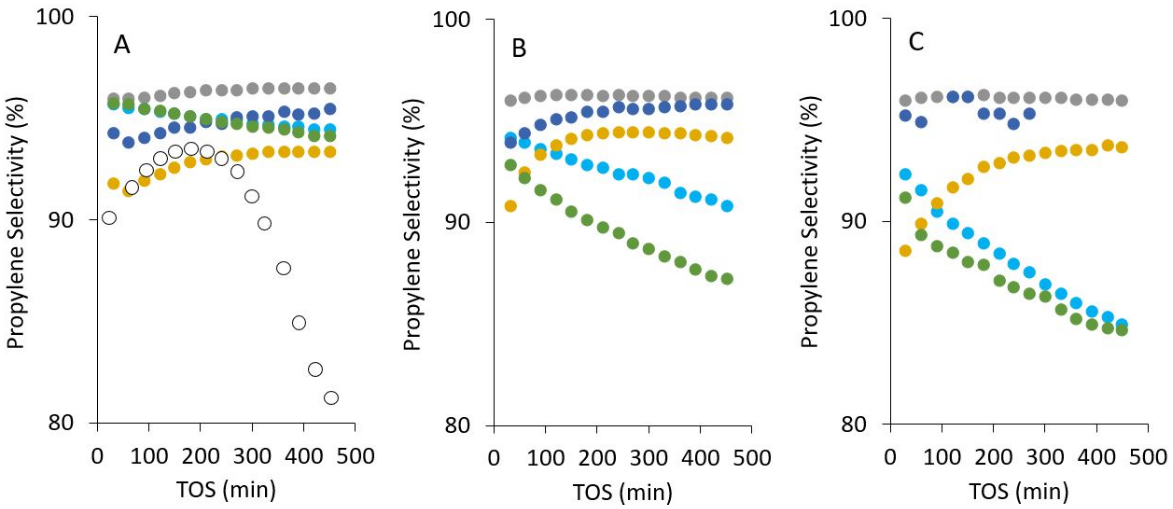

Figure S12: STEM images of the fresh PtSn/SiO_2 catalyst (a) and after two consecutive PDH reaction cycles (b). Reaction conditions: WHSV = 17.3 h^{-1} (on a propane basis); atmospheric pressure; 600°C; 3:1 N_2/propane molar ratio.

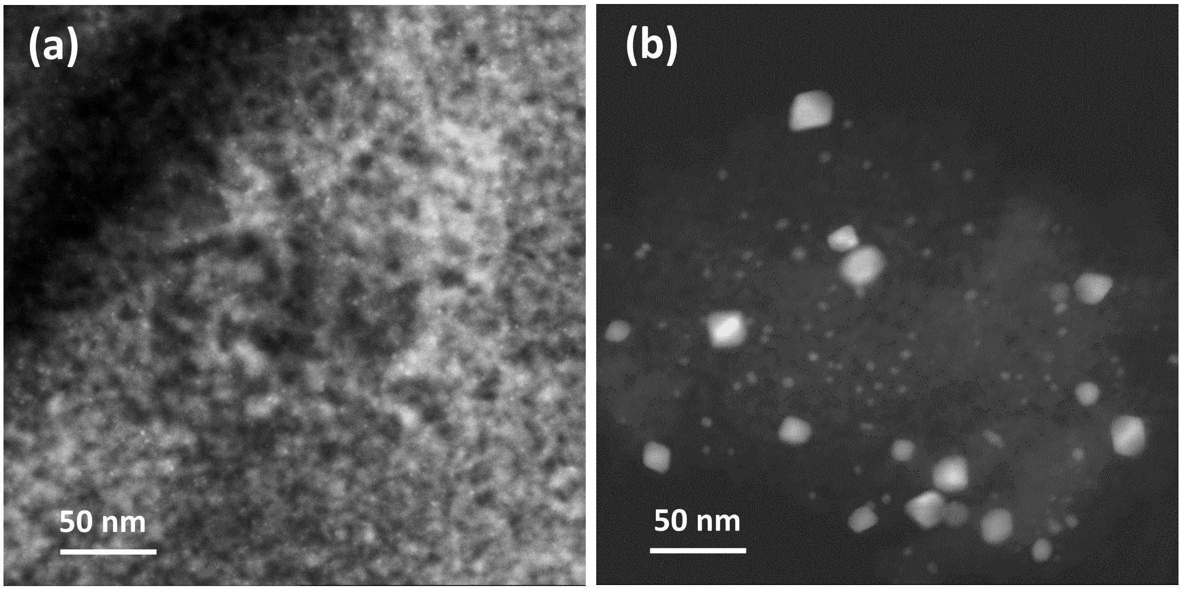

Figure S13: Propane conversion (blue) and propylene selectivity (red) for the PDH reaction using a highly concentrated propane stream for PtSn/SiO_2 (left) and MFI-PtSn9 (right) in their fresh forms (filled symbols) and after being regenerated (empty symbols) using a weight hourly space velocitie (WHSV) of 150 and 300 h^{-1} for the PtSn/SiO_2 and MFI-PtSn9, respectively. Reaction conditions: T = 550°C; P = 1 atm; feed gas, N_2 : propane = 1:9 (molar). After each cycle, the catalyst was regenerated by calcination in air at 600°C for 3 h.

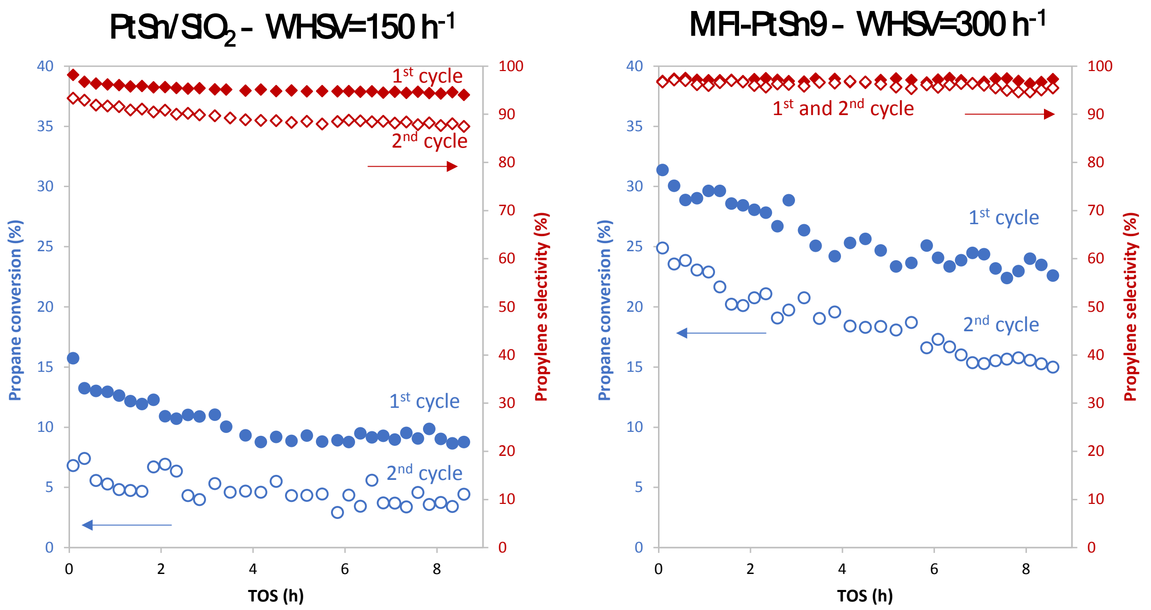

Figure S14: FESEM images of the MFI-PtSn9 and MFI-PtSn10 catalysts after three consecutive PDH reaction cycles. Reaction conditions: WHSV = 17.3 h^{-1} (on a propane basis); atmospheric pressure; 600°C; 3:1 N_2/propane molar ratio.

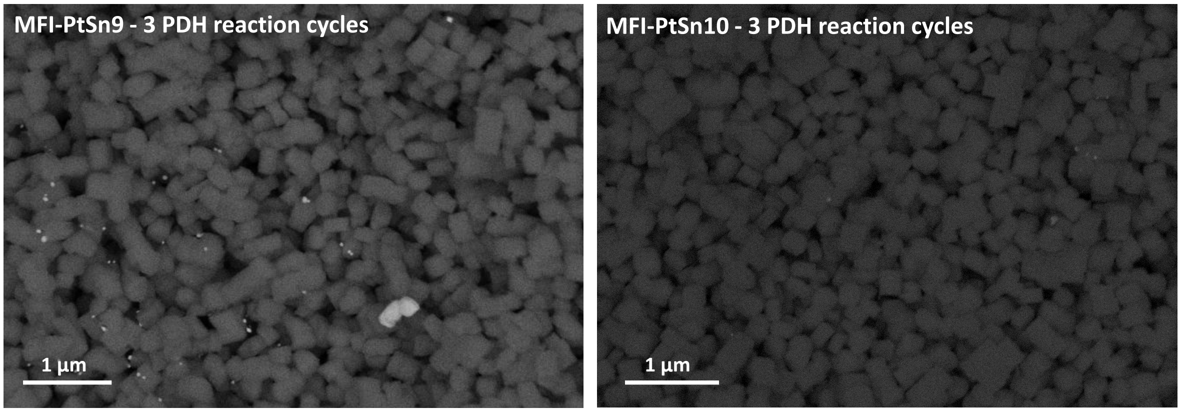

Figure S15: STEM images of the PtSn9 and PtSn10 materials after three consecutive PDH reaction cycles. Reaction conditions: WHSV = 17.3 h^{-1} (on a propane basis); atmospheric pressure; 600°C; 3:1 N_2/propane molar ratio.

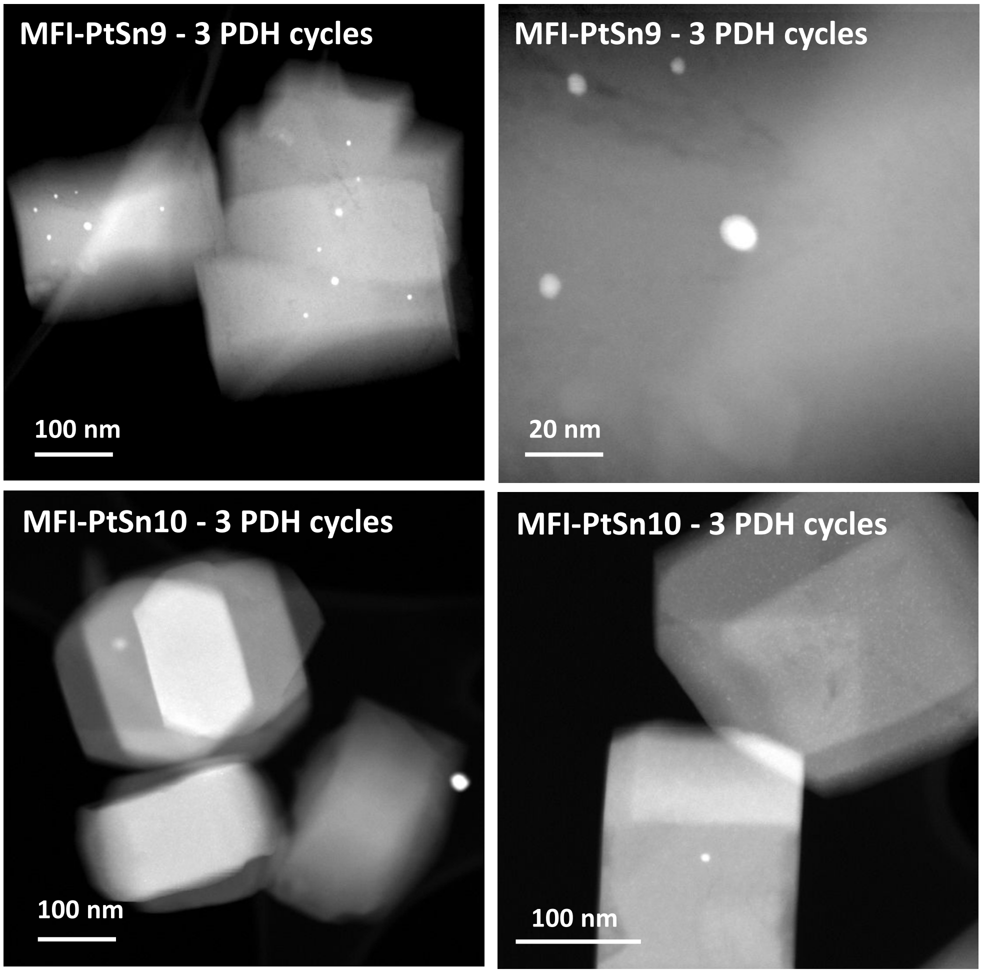

Figure S16. (A) Propane conversion as a function of the time-on-stream for the direct propane dehydrogenation reaction using MFI-PtSn9 (blue), MFI-PtSn10 (yellow) and MFI-PtSn11 (orange), at constant Pt and K contents of ~0.4 wt % Pt and ~0.6 wt % K, and variable Sn content of ~1, ~0.5, and ~0.2 wt % Sn, respectively, after calcination in air and reduction in H_2 for 1 h at 600°C. (B) Propane conversion as a function of the time-on-stream for the direct propane dehydrogenation reaction using the MFI-PtSn10 reduced in H_2 at 600°C for 1h (solid yellow) and 12 h (empty symbols) Reaction conditions: WHSV = 17.3 h^{-1} (on a propane basis); atmospheric pressure; 600°C; 3:1 N_2/propane molar ratio.

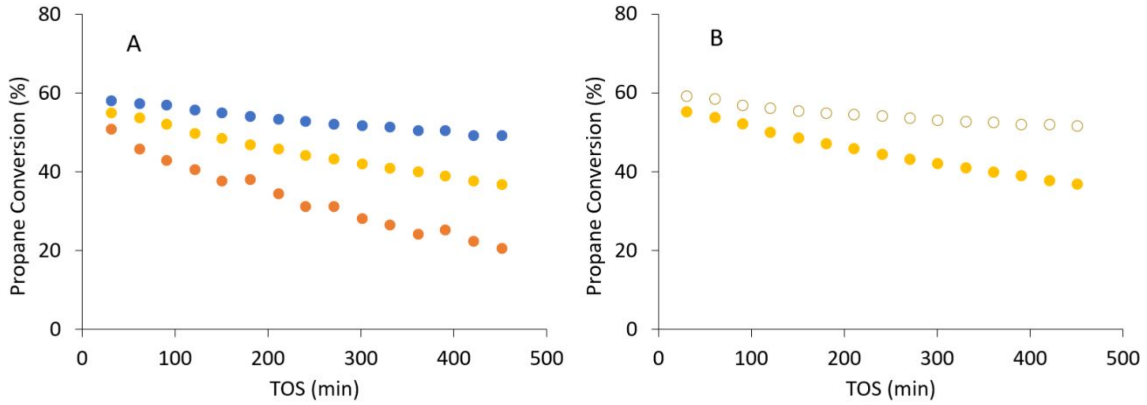

Figure S17. R space (magnitude and real parts of the Fourier Transform) and K space EXAFS data at k-weighting = 2 for samples MFI-PtSn9 (reduced 1 h in H_2), MFI-PtSn10 (reduced 1 h in H_2), MFI-PtSn10 (reduced 12 h in H_2), and MFI-PtSn9 (PDH spent), and the corresponding fittings using the parameters in Table 3. Blue lines are the experimental data, red lines are the corresponding fits.

MFI-PtSn9 (reduced 1 h in H_2)

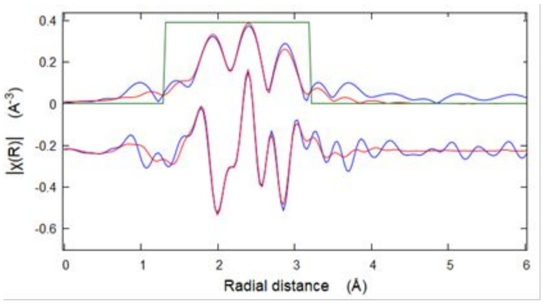

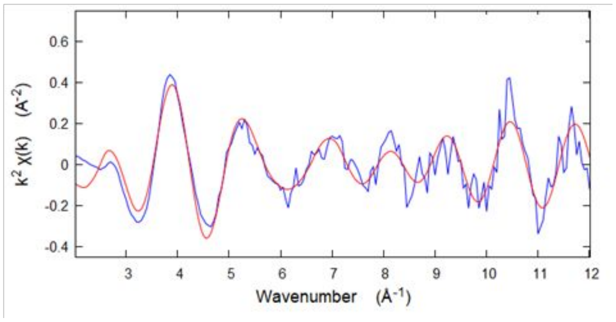

MFI-PtSn10 (reduced 1 h in H_2)

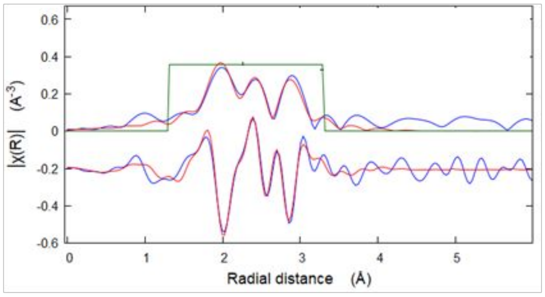

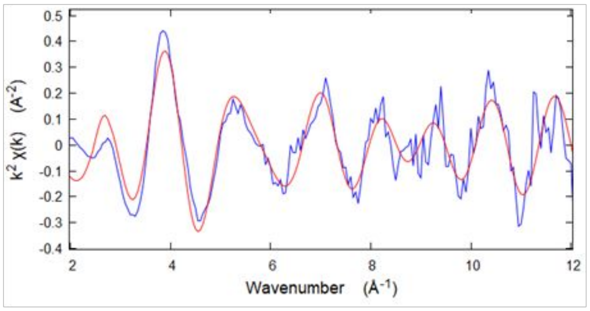

MFI-PtSn10 (reduced 12 h in H_2)

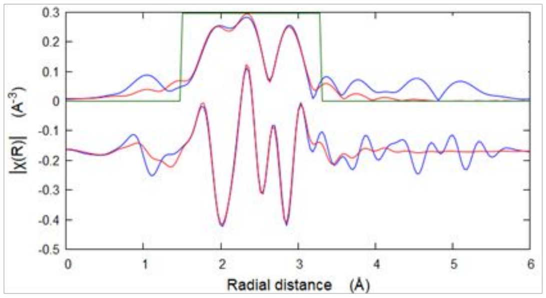

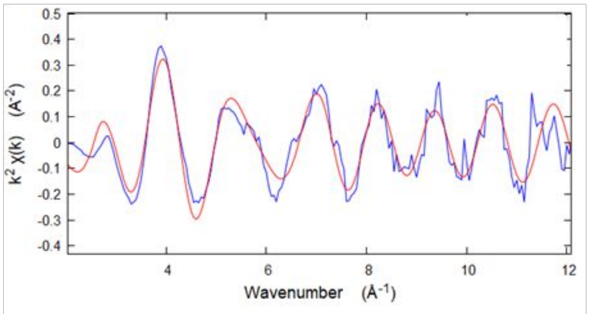

MFI-PtSn9 (PDH Spent)

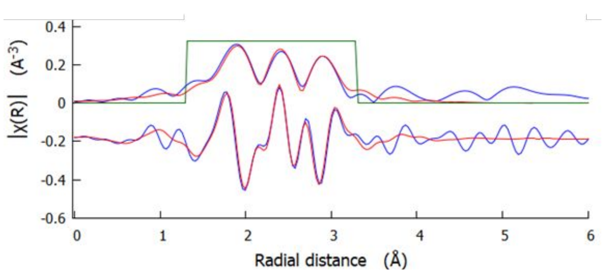

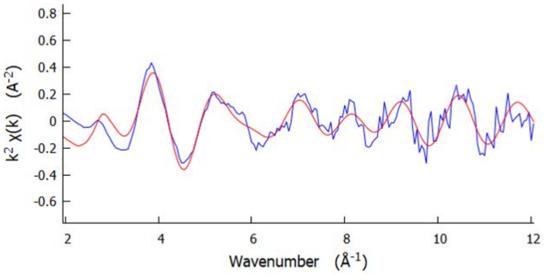

Supplementary Tables

Table S1: Relative stability of six MFI-TPA models containing four TPA molecules and the four associated silanol/siloxy nests placed in different locations around the channel's intersections of the MFI unit cell.

Model	E_{rel} (kcal/mol)
MFI-TPA-D1	0.0
MFI-TPA-D2	10,0
MFI-TPA-D3	11,4
MFI-TPA-D4	15,2
MFI-TPA-D5	17,0
MFI-TPA-D6	16,6

Table S2: Elemental and thermogravimetric analyses of the K-MFI sample.

Sample	C (wt.%)[a]	N (wt.%)[a]	C/N (molar ratio)	TGA weight loss (wt.%)[b]	TPA/u.c[c]	K/u.c.
MFI-7	9.19	0.89	12.0	13.4	4.10	1.02

[a] Calculated from the elemental analysis; [b] Calculated from the thermogravimetric analysis (TGA) up to 600°C; [c] u.c.: unit cell of MFI (96 T atoms)

Table S3: Optimized values of the Si-Si and K-O distances (in Å) involved in the incorporation of K in models F and H.

Label	Composition	rSi-Si(F)	rSi-Si(H)	rK-O(F)	rK-O(H)
MFI	$Si_{96}O_{192}$	3.196	3.201		
MFI-D1	$Si_{92}O_{192}H_{16}$	3.209	3.178		
MFI-D1-K	$Si_{92}O_{193}H_{17}K_1$	2.962	2.787		
MFI-4TPA	$Si_{92}O_{192}N_4C_{48}H_{124}$	3.208	3.190		
MFI-4TPA-K	$Si_{92}O_{193}N_4C_{48}H_{125}K_1$	2.995	2.802	2.964	2.654
MFI-4TPA-2K	$Si_{92}O_{194}N_4C_{48}H_{126}K_2$	3.002	2.792	2.926	2.623

References

(1) Motagamwala, A. H.; Almallahi, R.; Wortman, J.; Igenegbai, V. O.; Linic, S. Stable and Selective Catalysts for Propane Dehydrogenation Operating at Thermodynamic Limit. *Science (1979)* **2021**, *373* (6551), 217–222. https://doi.org/10.1126/science.abg7894.

(2) Ravel, B.; Newville, M. ATHENA, ARTEMIS, HEPHAESTUS: Data Analysis for X-Ray Absorption Spectroscopy Using IFEFFIT. *J Synchrotron Radiat* **2005**, *12* (4), 537–541. https://doi.org/10.1107/S0909049505012719.

(3) Zabinsky, S. I.; Rehr, J. J.; Ankudinov, A.; Albers, R. C.; Eller, M. J. Multiple-Scattering Calculations of x-Ray-Absorption Spectra. *Phys Rev B* **1995**, *52* (4), 2995–3009. https://doi.org/10.1103/PhysRevB.52.2995.

(4) Perdew, J. P.; Wang, Y. Accurate and Simple Analytic Representation of the Electron-Gas Correlation Energy. *Phys Rev B* **1992**, *45* (23), 13244–13249. https://doi.org/10.1103/PhysRevB.45.13244.

(5) Perdew, J. P.; Chevary, J. A.; Vosko, S. H.; Jackson, K. A.; Pederson, M. R.; Singh, D. J.; Fiolhais, C. Atoms, Molecules, Solids, and Surfaces: Applications of the Generalized Gradient Approximation for Exchange and Correlation. *Phys Rev B* **1992**, *46* (11), 6671–6687. https://doi.org/10.1103/PhysRevB.46.6671.

(6) Kresse, G.; Furthmüller, J. Efficient Iterative Schemes for Ab Initio Total-Energy Calculations Using a Plane-Wave Basis Set. *Phys Rev B* **1996**, *54* (16), 11169–11186. https://doi.org/10.1103/PhysRevB.54.11169.

(7) Blöchl, P. E. Projector Augmented-Wave Method. *Phys Rev B* **1994**, *50* (24), 17953–17979. https://doi.org/10.1103/PhysRevB.50.17953.

(8) Grimme, S. Semiempirical GGA-type Density Functional Constructed with a Long-range Dispersion Correction. *J Comput Chem* **2006**, *27* (15), 1787–1799. https://doi.org/10.1002/jcc.20495.

(9) Goerigk, L.; Grimme, S. A Thorough Benchmark of Density Functional Methods for General Main Group Thermochemistry, Kinetics, and Noncovalent Interactions. *Physical Chemistry Chemical Physics* **2011**, *13* (14), 6670. https://doi.org/10.1039/c0cp02984j.

(10) Grimme, S.; Antony, J.; Ehrlich, S.; Krieg, H. A Consistent and Accurate *Ab Initio* Parametrization of Density Functional Dispersion Correction (DFT-D) for the 94 Elements H-Pu. *J Chem Phys* **2010**, *132* (15). https://doi.org/10.1063/1.3382344.

(11) Grimme, S.; Ehrlich, S.; Goerigk, L. Effect of the Damping Function in Dispersion Corrected Density Functional Theory. *J Comput Chem* **2011**, *32* (7), 1456–1465. https://doi.org/10.1002/jcc.21759.

(12) Burton, A.; Terefenko, E.; Wang, H.; Paccagnini, M.; Sattler, A. Structure-Property Relationships That Influence Platinum Stability in All-Silica or Highly Siliceous Zeolites. *Microporous and Mesoporous Materials* **2023**, *350*, 112411. https://doi.org/10.1016/j.micromeso.2022.112411.
